# Thermochronological insights into reactivation of a continental shear zone in response to Equatorial Atlantic rifting (northern Ghana)

**DOI:** 10.1038/s41598-018-34769-x

**Published:** 2018-11-09

**Authors:** Nicholas Fernie, Stijn Glorie, Mark W. Jessell, Alan S. Collins

**Affiliations:** 1 Centre for Tectonics, Resources and Exploration (TRaX), Department of Earth Sciences, School of Physical Sciences, The University of Adelaide, Adelaide, SA-5005 Australia; 2 Centre for Exploration Targeting, School of Earth and Environment, The University of Western Australia, Crawley, WA 6009 Australia

## Abstract

West Africa was subjected to deformation and exhumation in response to Gondwana break-up. The timing and extent of these events are recorded in the thermal history of the margin. This study reports new apatite fission track (AFT) data from Palaeoproterozoic basement along the primary NE-SW structural trend of the Bole-Nangodi shear zone in northwestern Ghana. The results display bimodality in AFT age (populations of ~210-180 Ma and ~115-105 Ma) and length distributions (populations of 12.2 ± 1.6 and 13.1 ± 1.4 µm), supported by differences in apatite chemistry (U concentrations). The bimodal AFT results and associated QTQt thermal history models provide evidence for multiple cooling phases. Late Triassic – Early Jurassic cooling is interpreted to be related with thermal relaxation after the emplacement of the Central Atlantic Magmatic Province (CAMP). Early to middle Cretaceous cooling is thought to be associated with exhumation during the Cretaceous onset of rifting between West Africa and Brazil. Late Cretaceous – Cenozoic cooling can be related with exhumation of the Ivory Coast – Ghana margin and NNW-SSE shortening through western Africa. Furthermore, our data record differential exhumation of the crust with respect to the Bole-Nangodi shear zone, preserving older (CAMP) cooling ages to the south and younger (rifting) cooling ages to the north of the shear zone, respectively. This suggests that the Palaeoproterozoic BN shear zone was reactivated during the Cretaceous as a result of deformation in the Equatorial Atlantic region of Africa.

## Introduction

**Figure 1:**
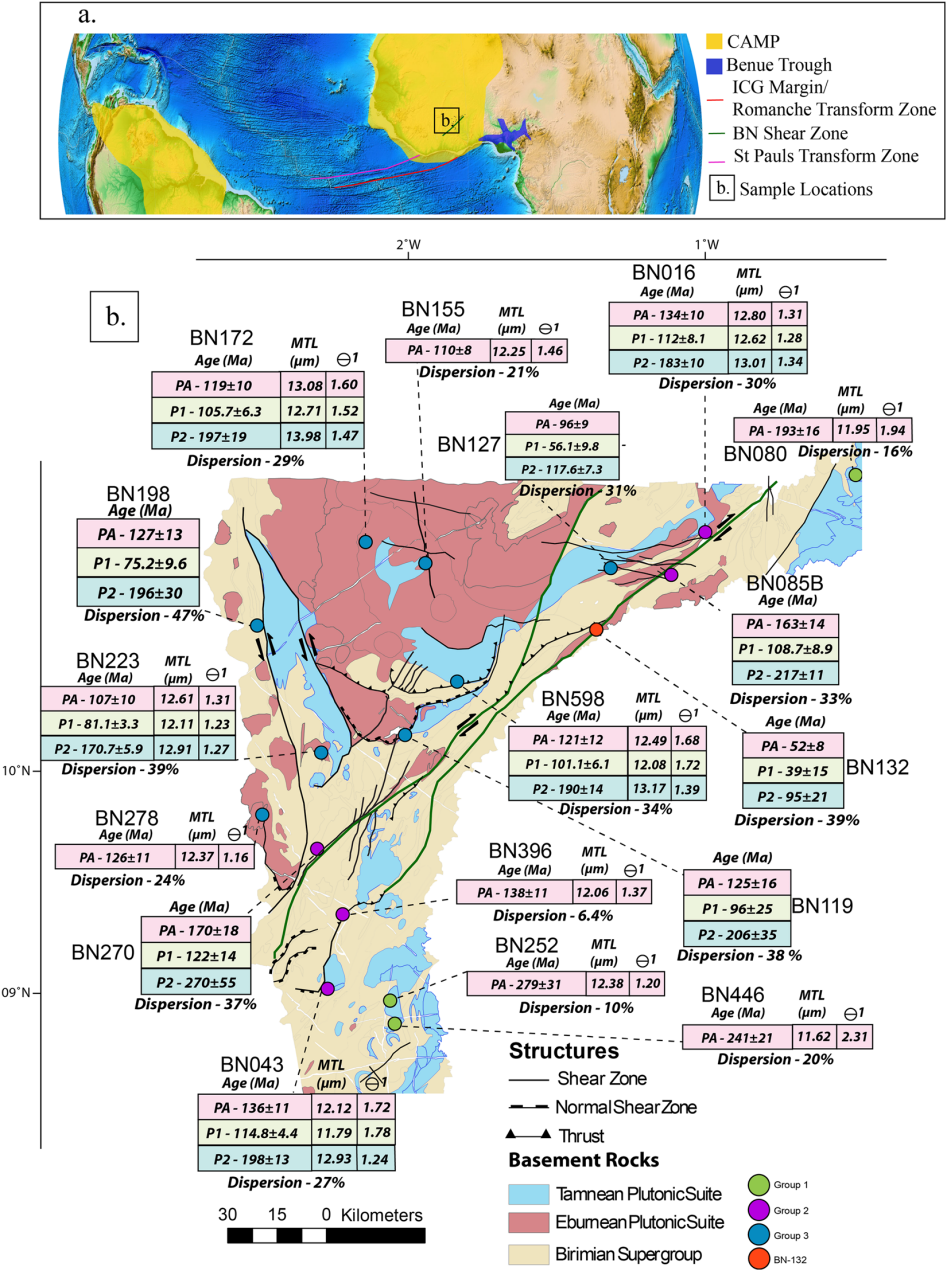
(**a**) Globe image with indication of the study area in Ghana (box framed) and showing the approximate locations of CAMP, the Benue Trough^17^, Ghana, the Bole-Nangodi shear zones and the Romanche and St Paul’s Transform Zones^12,19^. Modified from^61^ utilizing plate rotation files available in the Gplates Software Package version 1.5.0. https://www.gplates.org/. (**b**) Schematic geological map of the study area located in northern Ghana showing sample locations as coloured circles, lithologies and AFT results. PA – Pooled Age, P1 – Peak 1 Age, P2 – Peak 2 Age, MTL – Mean Track Length and ϴ1 – Standard Deviation of the track length distributions. Modified after^3^ utilizing ArcGIS Software Package version 10.3.1 http://desktop.arcgis.com/en/arcmap/.

The NE-SW striking Bole-Nangodi (BN) shear zone in northwestern Ghana represents a Palaeoproterozoic crustal-scale shear zone within the West African Craton (WAC)^1,2^ (Fig. 1) that preserves evidence for a deformation overprint that coincides with the Palaeoproterozoic Eburnean Orogeny^3^. The BN shear zone also contains structural evidence for subsequent distinct deformation phases^3^, however, the absolute timings of these brittle deformation phases are unknown. This study aims to characterise the Phanerozoic reactivation history of the shear zone and its role in the exhumation history of NW Ghana.

South of, and parallel to, the BN shear zone, the Ivory Coast-Ghana (ICG) continental margin forms a NE-SW trending basement ridge, which represents the eastern prolongation of the Romanche Transform Zone in the equatorial Atlantic Ocean (Fig. 1a)^4^. Geochronological and thermochronological studies have revealed that the ICG marginal ridge formed during the Cretaceous in response to rifting between the WAC and Brazil during the opening of the equatorial Atlantic Ocean^5–8^. Recent studies^9^ proposed that NE-SW oriented onshore faults in Ghana and the Ivory Coast are linked to an oceanic fracture zone 90 km South of the St Pauls transform zone in the Atlantic Ocean and that the eastern limit of the St Pauls Transform Zone can be linked with onshore faults in Ghana^10^. It is further suggested that the pre-existing shear zone may have controlled the orientation of the present-day oceanic transform zone^9^, however the theory remains inconclusive. Hence, this study hypothesises that the BN shear zone shares a common low-temperature thermo-tectonic history with the ICG marginal ridge. We present Apatite Fission Track (AFT) thermochronological data and associated thermal history models to constrain the low-temperature thermal and exhumation history of the BN shear zone, aiming to provide temporal constraints on the rifting history of the WAC from Brazil in response to the opening of the Equatorial Atlantic Ocean. Furthermore, we attempt to constrain the timing of shear zone reactivation, which has economic relevance to the exhumation and preservation of Ghana’s gold deposits^11,12^.

## Geological Setting

The study area in northern Ghana forms part of the south-eastern section of the WAC (Fig. 1)^12^. The WAC covers a large area across much of western Africa and consists of two Archean terranes in the north-western and south-western parts of the craton^1^. These Archean terranes are juxtaposed against an array of Palaeoproterozoic terranes that are made up of greenstone belts, contemporaneous volcano-sedimentary depocentres and regions of extensive tonalite-trondhjemite-granodiorite plutons, collectively known as the Tamnean plutonic suite, the Eburnean plutonic suite and the Birimian plutonic supergroup (emplaced between 2250 Ma and 1980 Ma)^3,4^. These terranes are overlain by Mesoproterozoic and younger sedimentary basins^2^, such as the Neoproterozoic to lower Palaeozoic Volta Basin^13^.

The WAC is considered to have been largely tectonically inactive from the Gondwana-forming orogeny until the Triassic-Jurassic transition at ca. 200 Ma^9^. The time period between the end of the Triassic and the early Cretaceous is characterised by two stages of rifting and related magmatism that precede the opening of the Equatorial Atlantic Rift System: (1) break-up within the Central Atlantic transpired at ca. 200 Ma, contemporaneous with the emplacement of the Central Atlantic Magmatic Province (CAMP)^14,15^ and (2) break-up within the South Atlantic occurred synchronously or marginally preceded the emplacement of the Paraná – Etendeka Large Igneous Province (LIP) at ca. 137 Ma as dated by ^40^Ar-^39^Ar methods^14–16^. Furthermore, magmatism in the Benue Trough between 147 and 106 Ma (as dated by ^40^Ar-^39^Ar methods) preceded the final stages of break-up in in the equatorial Atlantic Ocean and overlaps in time with Paraná – Etendeka magmatism^14,17^. Rocks related to those igneous events are limited within Ghana and are mostly constrained to Nigeria, Guinea, Mali, Morocco, Mauritania and Liberia within the African continent^18,19^. Crossing the Atlantic from the Ghana study area, the Maranhão and Parnaíba Basins in north-eastern Brazil (Fig. 1a) yield abundant volcaniclastic evidence of both CAMP and Paraná – Etendeka magmatism (based on geochemical associations)^16^. Both the African and Brazilian margins, thus, contain evidence for rifting of the Atlantic Ocean over a 70–80 myr. period^17,20–22^.

The ICG marginal ridge is the modern expression of the Romanche transform fault associated with rifting and break-up in the equatorial Atlantic during the Early Cretaceous^23^. Off-shore, low-temperature thermochronological and geophysical studies conducted on the ICG margin reveal its development to have undergone three geodynamic stages since the Aptian^5,6,8,10,24^. (1) At the start of the Aptian, the first stages of continental rifting and transform faulting occurred and the Deep Ivorian Basin was initiated as a result of E-W to ENE-WSW oblique extension^5,10^ (2) Intra-continental transform faulting between the African and South American plates led to other rapidly forming isolated rift basins (eg. Tano Basin) north of the transform during the late Aptian to Cenomanian^5,6,10^. During this time, the ICG margin experienced a shift from an overall transtensional to a transpressional tectonic setting as a result of a change in the orientation of drift in the South American plate from SW to SSW^10,25^. The change in drift direction is recorded in a series of folds and flower structures in offshore basins north of the margin^10^. This short period (between the late Albian and Cenamonian) of intense rifting and deformation is marked by a pronounced angular unconformity associated with basin inversion across the southern edge of the margin and exhumation of the outer ridge. Deformation during this time is thought to mark the final stages of contact between the rifting continents^10^. (3) During the Turonian, continued transform faulting resulted in the transition to seafloor spreading and caused newly created oceanic crust to come into contact with the continent. The mechanism for the cooling of the ICG margin is debated.^6^ interprets the late Cretaceous cooling to be related with exhumation in response to the bathymetric step between the continental margin and the oceanic crust, while^4,5^ interpret the cooling signal to be a flexural response to unloading during continental separation. Since the Santonian, the ICG margin became passive^4,6,8^. Furthermore, at the end of the Cretaceous (~65 Ma), western Africa was experiencing NNW-SSE shortening, folding and strike-slip faulting, which induced an angular unconformity in the Benue Trough^26,27^.

Within the study area, seven relative deformation events are described, for which absolute ages are only recorded related with D1, D2 and D3. These early deformation events are interpreted to be associated with the Palaeoproterozoic Eburnean Orogeny^3,28,29^. Later deformation events in the area are described as localised E-W to NE-SW striking brittle deformation associated with dextral strike-slip shearing under a transcurrent regime^3^. Prior to this study, the timings of these fault events are only relatively constrained.

## Results

### Sample Locations

A total of 17 samples were chosen based on apatite quality and on their proximity to the BN shear zone in northern Ghana (Fig. 1b). All samples were sourced from crystalline rock types and metamorphic equivalents. Sample locations and rock descriptions are detailed in Table 1 and locations are shown in Fig. 1. The resulting AFT results are grouped according to their location relative (northwest, southeast and proximity) to the BN shear zone (Fig. 1b) and age, allowing assessment of differential cooling across the study area with respect to the shear zone. Group 1 contains three samples BN-080, BN-252 and BN-446, located near the margin of the Volta Basin, to the southeast of the BN shear zone (Fig. 1b). Group 2 is composed of five samples BN-016, BN-043, BN-085B, BN-270 and BN-396, which were taken from within the BN shear zone or associated splay faults (Fig. 1b). Group 3 contains eight samples located to the northwest of the BN shear zone (BN-119, BN-127, BN-155, BN-172, BN-198, BN-223, BN-278, and BN-598) (Fig. 1b). One sample (BN-132) from within the shear zone was treated separately as it records younger AFT ages than any other sample. In general, AFT pooled ages range from 279 ± 31 Ma (oldest sample) to 52 ± 8 Ma (youngest sample) and associated Dpar measurments range from 2.94 ± 0.73 µm to 1.56 ± 0.17 µm (Table 2).

**Table 1:** Sample locations and lithology details. U-Pb formation ages, formation/suite and lithology’s are from^3,5,12^. Migm = migmatite.

Sample ID	Latitude	Longitude	Elevation	Igneous Suites	Lithology	Published U-Pb Formation Age (Ma)
*BN-016*	10°48′18.00″N	0°53′6.00″W	216 m	Birimian Super Group	Granite	2250-1980
*BN-043*	8°55′44.04″N	2°25′55.20″W	277 m	Tamnean Pluton Suite	Granite	2134-2118
*BN-080*	11°2′24.00″N	0°16′40.80″W	219 m	Tamnean Pluton Suite	Granite	2134-2118
*BN-085B*	10°37′55.20″N	1°1′55.20″W	165 m	Birimian Super Group	Granite	2250-1980
*BN-119*	9°58′9.12″N	2°6′54.00″W	307 m	Tamnean Plutonic Suite	Granite	2134-2118
*BN-127*	10°39′10.80″N	1°16′19.20″W	181 m	Eburnean Plutonic Suite	Bt-Gneiss	2150-2000
*BN-132*	10°24′3.60″N	1°19′55.20″W	162 m	Eburnean Plutonic Suite	Orthogneiss	2150-2000
*BN-155*	10°40′22.80″N	2°1′51.60″W	285 m	Eburnean Plutonic Suite	Orthogneiss	2150-2000
*BN-172*	10°45′36.00″N	2°16′37.20″W	306 m	Eburnean Plutonic Suite	Bt-Granite	2150-2000
*BN-198*	10°25′1.20″N	2°43′15.60″W	282 m	Birimian Super Group	Granite	2250-1980
*BN-223*	9°53′53.16″N	2°27′28.80″W	324 m	Eburnean Plutonic Suite	Bt-Msc-Granite	2150-2000
*BN-252*	8°52′50.16″N	2°10′33.60″W	241 m	Tamnean Plutonic Suite	Granite	2134-2118
*BN-270*	9°30′27.00″N	2°27′54.00″W	292 m	Birimian Super Group	Orthogneiss	2250-1980
*BN-278*	9°32′35.88″N	2°42′0.00″W	293 m	Eburnean Plutonic Suite	Bt-Msc-Granite	2150-2000
*BN-396*	9°14′4.56″N	2°22′12.00″W	298 m	Birimian Super Group	Paragneiss	2250-1980
*BN-446*	8°47′10.32″N	2°9′25.20″W	222 m	Birimian Super Group	Paragneiss	2250-1980
*BN-598*	10°11′20.40″N	1°54′3.60″W	248 m	Tamnean Plutonic Suite	Granodiorite	2134-2118

**Table 2:** AFT dating results organised according to groupings. ρ_s_ is the average surface density of spontaneous fission tracks (in 10^5^ tracks/cm^2^). Ns is the total number of counted spontaneous tracks. n is the number of grains analysed.

Sample ID	ρ_s_ (×10^5^)	Νs	n	^238^U	1 σ	P. Age (Ma)	1 σ	C. Age (Ma)	1 σ	P_1_ (Ma)	1 σ	P_2_ (Ma)	1 σ	Disp (%)	P(χ²)	nl	MTL (µm)	SD	Dpar (µm)	SD
*BN-080*	4.3	608	41	4.5	0.3	193	16	200	10	—	—	—	—	16	0.07	54	11.95	1.94	2.31	0.63
*BN-252*	6.7	418	28	4.7	0.5	279	31	298	18	—	—	—	—	10	0.25	31	12.38	1.36	1.58	0.30
*BN-446*	19.2	997	26	14.7	1.2	241	21	231	13	—	—	—	—	20	0.00	35	11.62	2,31	1.70	0.29
*BN-016*	12.5	2952	48	16.9	1.3	134	10	146	7	112	8	183	10	30	0.00	69	12.80	1.32	1.96	0.17
*BN-043*	23.3	1974	41	30.5	2.5	136	11	132	7	115	4	198	13	27	0.00	60	12.12	1.72	2.26	0.37
*BN-085B*	6.4	890	40	7.6	0.6	163	14	174	11	109	9	217	11	33	0.00	—	—	—	2.00	0.40
*BN-396*	20.3	1916	39	26.1	2.1	138	11	138	4	—	—	—	—	6.4	0.10	87	12.06	1.38	1.56	0.17
*BN-270*	3.0	310	29	3.4	0.3	170	18	152	15	122	14	270	55	37	0.00	—	—	—	1.99	0.45
*BN-172*	7.9	814	41	11.4	0.9	119	10	129	8	106	6	197	19	29	0.00	55	13.08	1.60	2.94	0.73
*BN-198*	5.3	136	9	9.5	0.8	127	13	116	22	75.2	10	196	30	56	0.00	—	—	—	2.16	0.67
*BN-223*	20.7	2486	38	39.0	3.5	107	10	127	9	81.1	3	171	6	39	0.00	70	12.61	1.31	1.73	0.23
*BN-278*	17.3	1415	30	25.4	2.2	126	11	122	7	—	—	—	—	24	0.00	36	12.37	1.16	2.21	0.39
*BN-598*	11.4	1317	36	15.7	1.5	121	12	128	10	101	6	190	14	34	0.00	64	12.49	1.68	2.61	0.42
*BN-155*	15.7	2008	41	32.2	2.2	110	8	98	4	—	—	—	—	21	0.00	142	12.25	1.47	2.55	0.20
*BN-119*	3.9	205	34	5.4	0.6	125	16	141	14	96	25	206	35	38	0.00	—	—	—	2.01	0.34
*BN-127*	9.0	468	26	18.3	1.6	96	9	102	8	56.1	10	118	7	31	0.00	—	—	—	2.34	0.45
*BN-132*	2.3	72	13	8.7	0.8	52	8	65	11	39	15	95	21	39	0.03	—	—	—	2.01	0.70

^238^U is the average ^238^U concentration, measured by LA-ICP-MS (in μg/g) with its uncertainty and calculated in Iolite following^55^. P. Age is the pooled AFT age in Ma, C. Age is the central AFT age in Ma^63^, statistically generated for each sample using Radialplotter^51^ (in Ma). P_1_ and P_2_ are statistically derived age populations in Radiaplotter^51^. Disp gives the percentage of single-grain age dispersion. P(χ^2^) is the chi-squared probability that the dated grains belong to a single statistical population (samples fail this test if P(χ^2^) <0.05). nl is the number of measured confined tracks. MTL is the average confined track length in μm with SD as the standard deviation of the distribution. Dpar is the average etch-pit diameter in μm with SD as the standard deviation of the distribution. We used Radialplotter software^51^ version 8.3, http://www.ucl.ac.uk/~ucfbpve/radialplotter/.

### Group 1: southeast of the BN shear zone

**Figure 2:**
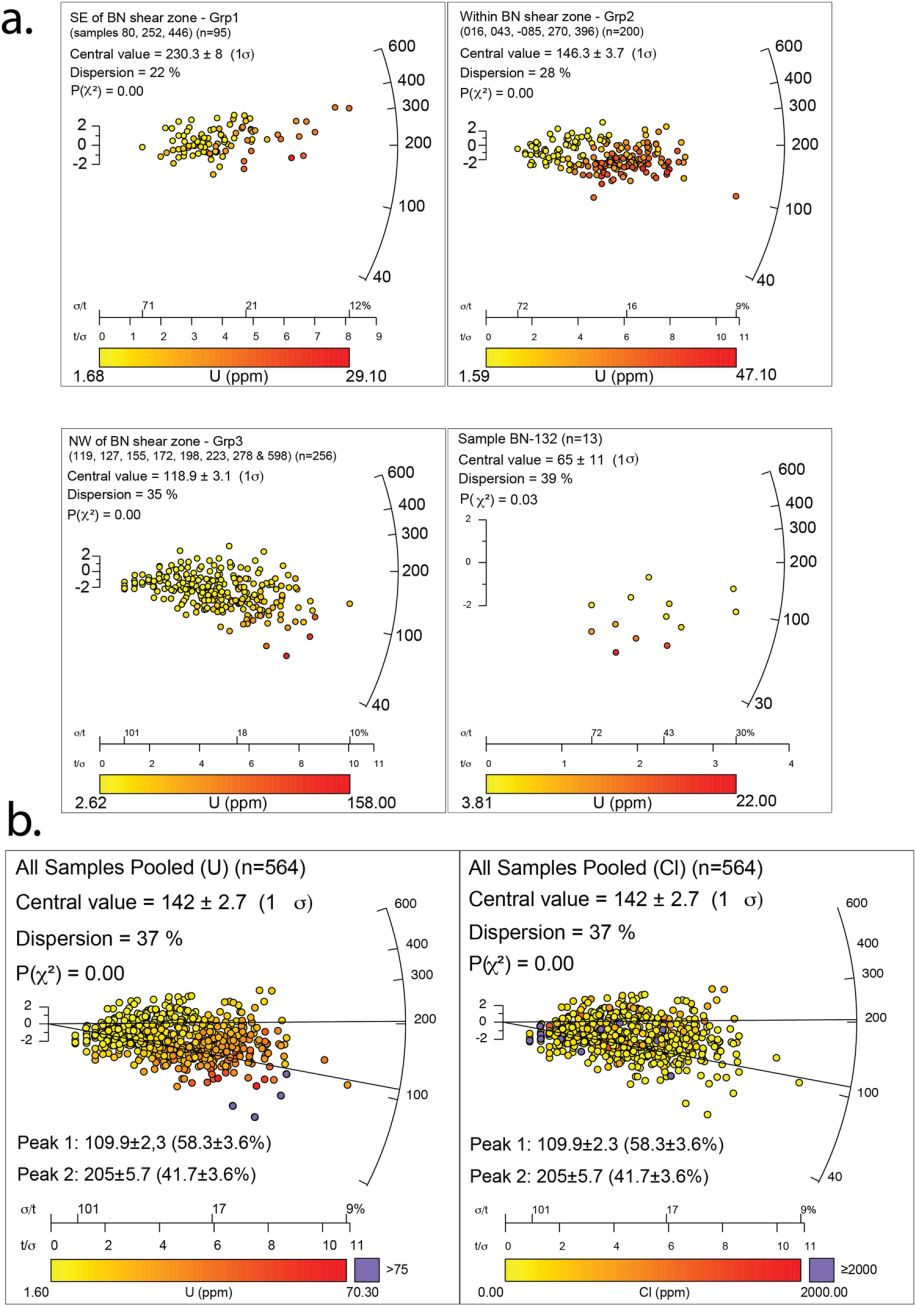
AFT results plotted as radial plots. Central age values were calculated using RadialPlotter and for dispersions >25% and P(χ^2^) less than 0.05 age peak discrimination was performed using the RadialPlotter software^51^ version 8.3 http://www.ucl.ac.uk/~ucfbpve/radialplotter/. Percentage of data associated with each peak is bracketed adjacent to the age of each peak. U (ppm) on the X-axis represents the amount of ^238^U in ppm. The left Y-axis represents 2 standard deviations from the central age. The right ‘curved’ Y-axis shows increasing age in Ma. The values along the X-axis show single-grain age uncertainties. (**a**) Radial plots of calculated AFT cooling ages for each sample location pooled into three sample groups: ‘SE of BN shear zone’, ‘within BN shear zone’ and ‘NW of BN shear zone’. Sample BN-132 (the youngest sample of the study area) is shown separately. (**b**) Radial plots of calculated AFT cooling ages for each sample location pooled together. Left: Pooled data where single grain ages are discriminated according to ^238^U concentration. Right: Pooled data where single grain ages are discriminated according to Cl concentration. These plots reveal that apatites associated with P1 yield on average lower U and higher Cl concentrations than those associated with P2, suggesting a chemical control on fission track annealing.

Group 1 (referred to as SE of BN shear zone) yield the oldest AFT results for this study. The pooled AFT ages obtained for individual samples in this Group range between 193 ± 16 Ma to 279 ± 31 Ma (Fig. 1b; Table 2; Supplementary Fig. S1). Samples BN-252 and BN-446 were taken in the south-western corner of the study area and revealed pooled AFT ages of 279 ± 31 Ma and 241 ± 21 Ma and mean track lengths (MTL) of 12.38 µm and 11.62 µm respectively (Table 2). Sample BN-080 was taken within the north-eastern corner of the study area, and has a pooled AFT age of 193 ± 16 Ma with a MTL of 11.95 µm. Dpar values range from 1.58 ± 0.30 to 2.31 ± 0.63 µm. A pooled radial plot for Group 1 yields a central AFT age of 230 ± 8 Ma (Fig. 2a).

### Group 2: within the BN shear zone

Group 2 (referred to as ‘within BN shear zone’) yields younger central ages and relatively large single-grain age dispersion. The pooled AFT ages for this Group range between 134 ± 10 Ma and 170 ± 18 Ma (Fig. 2a; Table 2; Supplementary Fig. S2). BN-270 and BN-085B represent the oldest samples in the group with pooled AFT ages of 170 ± 18 Ma and 163 ± 14 Ma (Table 2). Samples BN-016, BN-043, BN-119 and BN-396 have largely consistent pooled AFT ages ranging between 134 ± 10 Ma and 141 ± 14 Ma. Three samples from this group contain sufficient confined lengths that could be measured for subsequent thermal history modelling. Sample BN-016 has a MTL of 12.80 µm, BN-043 has a MTL of 12.12 µm and BN-396 has a MTL of 12.06 µm (Table 2). All samples, except BN-396, failed the χ^2^ test and yield single grain dispersion values of >25%, suggesting a complex thermal history record. For those samples, two statistical age populations were derived (defined as peak ages P1 and P2). P1 ages range between 112 ± 8 and 122 ± 14 Ma. P2 ages cluster between 183 ± 10 and 217 ± 11 Ma, with one older exception (270 ± 55 Ma) (Table 2; Supplementary Fig. S2). Dpar values range from 1.56 ± 0.17 to 2.26 ± 0.37 µm. A pooled radial plot was constructed for the samples from within the BN shear zone, yielding a central AFT age of 146 ± 4 Ma (Fig. 2a). The plot shows a broad distribution of single grain ages and a trend of younger grains associated with higher uranium concentrations and vice versa.

### Group 3: northwest of the BN shear zone

Group 3 (referred to as ‘NW of BN shear zone’) samples exhibit pooled AFT ages ranging between 96 ± 9 Ma and 127 ± 13 Ma. Individual sample pooled AFT ages and MTL data can be found in Table 2. Most samples (except BN-155 and BN-278) fail the χ^2^ test and RadialPlotter discriminates two statistically probable age peaks (P1 and P2) for these samples, with P1 ranging from 75 ± 10 Ma to 106 ± 6 Ma and P2 ranging from 171 ± 6 Ma to 206 ± 35 Ma. Sample BN-127 acts as an exception and yields a P1 age of 56 ± 10 Ma and P2 age of 118 ± 7 Ma (Fig. 2a; Table 2; Supplementary Fig. S3). MTL data was obtained for five samples and range from 13.08 to 12.25 µm. Dpar values range from 1.73 ± 0.23 to 2.94 ± 0.73 µm (Table 2). The pooled radial plot yields a central AFT age of 119 ± 3 Ma. Similarly as for Group 2, discussed above, the youngest single-grain ages correlate with the highest uranium concentrations and vice versa.

### Sample BN-132

Sample BN-132 produced the youngest AFT age for the study area (52 ± 8 Ma). Sample BN-132 fails the χ^2^ test and RadialPlotter discriminates two statistically probable age peaks with a P1 of 39 ± 15 Ma and a P2 of 95 ± 21 Ma (Fig. 2a; Table 2; Supplementary Fig. S4). Similarly as the other sample groups, the youngest single-grain ages correlate with the highest uranium concentrations and vice versa.

## Interpretations

### Apatite Fission Track Thermal History Modelling

Thermal history (tT) modelling was conducted on eleven individual samples that yield sufficient confined lengths for modelling purposes (Table 2). Given that AFT was the sole low-temperature thermochronometer used in this study and no other published thermochronological data are available for the study area, modelling space was limited to the apatite partial annealing zone (~120°–60 °C) without forward modelling constraints. Interpretations of tT plots are based on the probability output, which acts as a weighted distribution for the most likely or ‘expected’ tT path. Note that the confidence interval of the probability distribution for several models is rather large and, therefore, for those models the magnitude and exact timing of cooling is not always well constrained.

**Figure 3:**
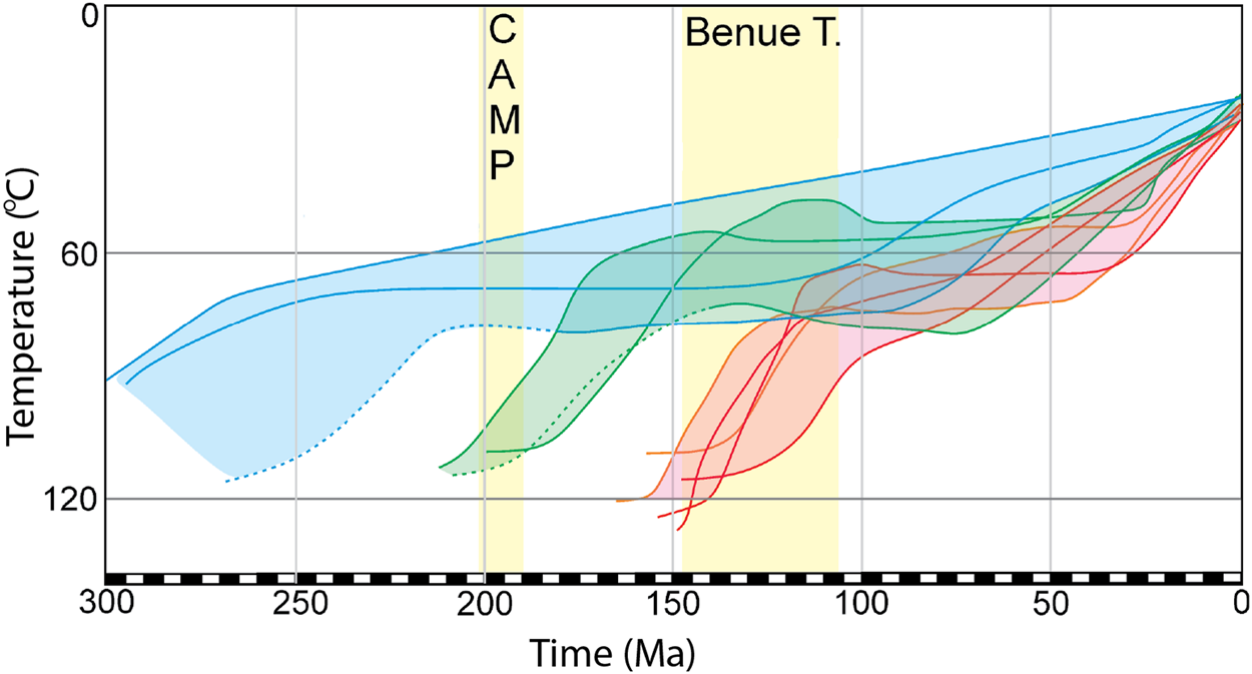
Thermal History models (tT) of all individually (11) modelled samples (Supplementary Fig. S5), overlain on the same plot. Thermal History models (tT) for all samples are based on AFT and MTL data from each individually modelled sample and developed using QTQt software^59^ version 4 http://www.iearth.org.au/codes/QTQt/. Constraints utilized for modelling are displayed in Supplementary Table 2. The left Y-axis represents temperature (°C). The X-axis represents Time (Ma). Blue tT paths represent samples from the SE of the BN Shear Zone, green tT paths represent samples from within the BN shear zone and red tT paths represent samples from the NW of the BN Shear Zone. CAMP = Central Atlantic Magmatic Province, Benue T. = Benue Trough.

The modelled samples display three distinct cooling periods (Fig. 3), which are defined by the three groups listed above. Models for samples in group ‘SE of BN shear zone’ exhibit rather slow cooling from the Triassic until present day temperatures. Models for samples in group ‘within the shear zone’ show fast cooling since the Late Triassic – Early Jurassic (ca. 200 Ma), followed by thermal quiescence until the end of the Cretaceous. The long residence of the samples in the APAZ during most of the Jurassic and Cretaceous likely explains the rather large single grain age dispersion observed for samples from within the BN shear zone (Fig. 2a). Since the start of the Cenozoic, the modelled tT history suggests increased cooling rates, bringing the samples above the APAZ during the Palaeogene (Fig. 3). Models for samples in group ‘NW of BN-shear zone’ exhibit fast cooling since the Late Jurassic – Early Cretaceous (ca. 150 Ma). Similar as for the samples from within the shear zone, thermal quiescence was observed, before the samples were brought to near-surface temperatures during the Palaeogene. The two samples that record the youngest age peaks for the study area (BN-127, P1 = ca. 56 Ma, BN-132 P1 = ca. 39 Ma) were not modelled due to insufficient available AFT length data. However, the P1 ages obtained for those samples coincides with the timing of the final cooling step in the thermal history models (ca. 60-35 Ma). Individual models, modelled track length distributions and modelling parameters can be found in Supplementary Fig. S5 and Supplementary Table 2.

In summary, a Late Triassic-Jurassic cooling signal and a middle Cretaceous cooling signal were modelled, consistent with the two distinct age populations. In addition, late Cretaceous - Palaeogene cooling is recorded for several thermal history models, confirmed by the youngest AFT age peaks in the study area. However, this modelled Palaeogene cooling signal could represent a modelling artefact, induced by constraining the model to present-day ambient temperatures, and therefore caution is required during interpretation in further discussion.

### The role of uranium on the recorded AFT data

The thermal history models display a rather complex thermal history record (Fig. 3) with evidence for multiple cooling phases. The identified AFT age-populations (i.e. P1 and P2) preserve different parts of the thermal history record (Table 2), suggesting that apatite grains associated with each population record cooling at different temperatures (i.e. upper and lower APAZ temperatures). In order to strengthen this observation, fission track length distributions were compared to AFT ages. If the different age-populations indeed preserved different parts of the thermal history, they could likely record different fission track length distributions.

**Figure 4:**
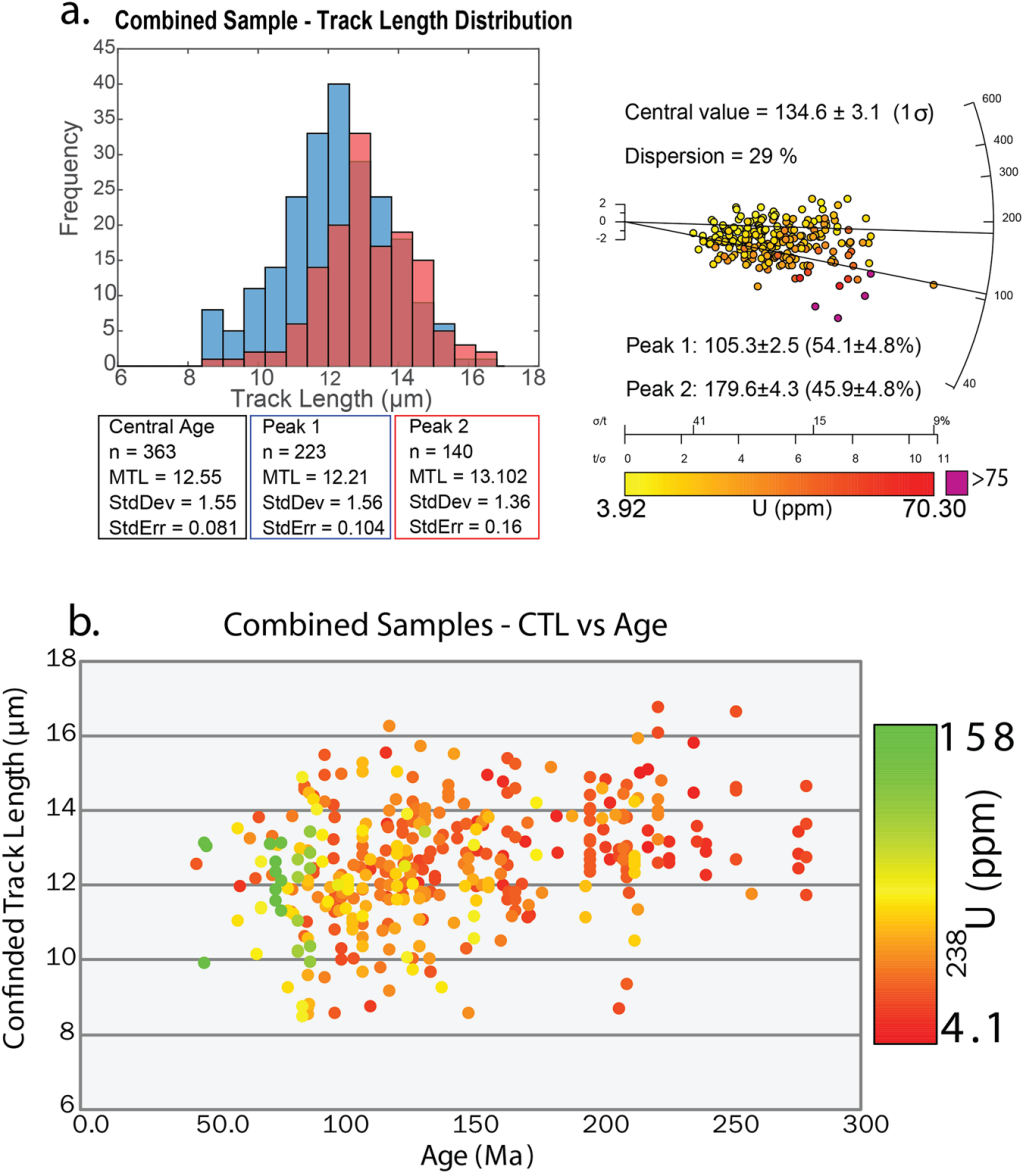
(**a**) Left: Frequency plots showing the confined track length (µm) distribution for Peak 1 and Peak 2 AFT age populations. Right: - Pooled radial plot for the samples used in the confined track length experiment. Central age values are calculated by RadialPlotter and for dispersions >25% age peak discrimination was performed using the RadialPlotter software^51^ version 8.3 http://www.ucl.ac.uk/~ucfbpve/radialplotter/. Percentage of data associated with each peak is bracketed adjacent to the age of each peak. The X-axis represents the amount of ^238^U in ppm. The left Y-axis represents 2 standard deviations from the central age (Ma). The right ‘curved’ Y-axis shows increasing age in Ma. ^238^U ppm values greater than 75 ppm are displayed as purple circles. (**b**) Pooled Cartesian plot showing the relationship between Age (Ma) on the X-axis, Confined track lengths (µm) on the Y-axis and ^238^U concentrations displayed as a colour gradient.

Five samples from within and NW of the shear zone were used for this exercise, where confined tracks were only measured in AFT dated grains. All samples yield high single grain dispersions and low P(χ2) values and exhibit clear open-jaw signatures in the radial plots (samples BN-016, BN-043, BN-172, BN-223 and BN-598). For each of those samples, confined track lengths related to AFT age populations P1 are displayed in a blue frequency plot and those related to AFT age populations P2 are displayed in a red frequency plot (Fig. 4a). The MTL for confined lengths recorded in apatites related with population P1 (based on 223 length measurements) is 12.21 ± 1.56 µm. The MTL for apatites associated with population P2 (based on 140 length measurements) is slightly higher at 13.10 ± 1.36 µm (Fig. 4a), suggesting that P1 grains experienced more extensive AFT annealing than P2 grains.

In addition, the relation between AFT age, confined track length and ^238^U concentration was evaluated (Fig. 4b). As shown, there is a positive correlation of P2 ages associated with longer confined track lengths and lower ^238^U concentrations, while younger (P1) ages correlate with shorter track lengths and higher ^238^U concentrations. There are no clear correlations observed between AFT ages and Cl concentrations^30^ (Fig. 2b), nor between AFT ages and Dpar measurements^31^ (Supplementary Fig. S6). We, therefore, suggest that the concentration of uranium controls AFT annealing in our study area. The relation between uranium concentration and AFT age has been observed for other, unrelated, study areas as well^32–34^. ^35^Suggests a model where elevated concentrations of Uranium lead to radiation enhanced annealing. More recently,^35^ observed, using transmission electron microscopy measurements, that alpha particle induced annealing of radiation damage indeed occurs in apatite. This study illustrates that the effect of radiation-induced annealing needs to be evaluated in fission track studies.

## Discussion

### Late Triassic to Early Jurassic cooling

**Figure 5:**
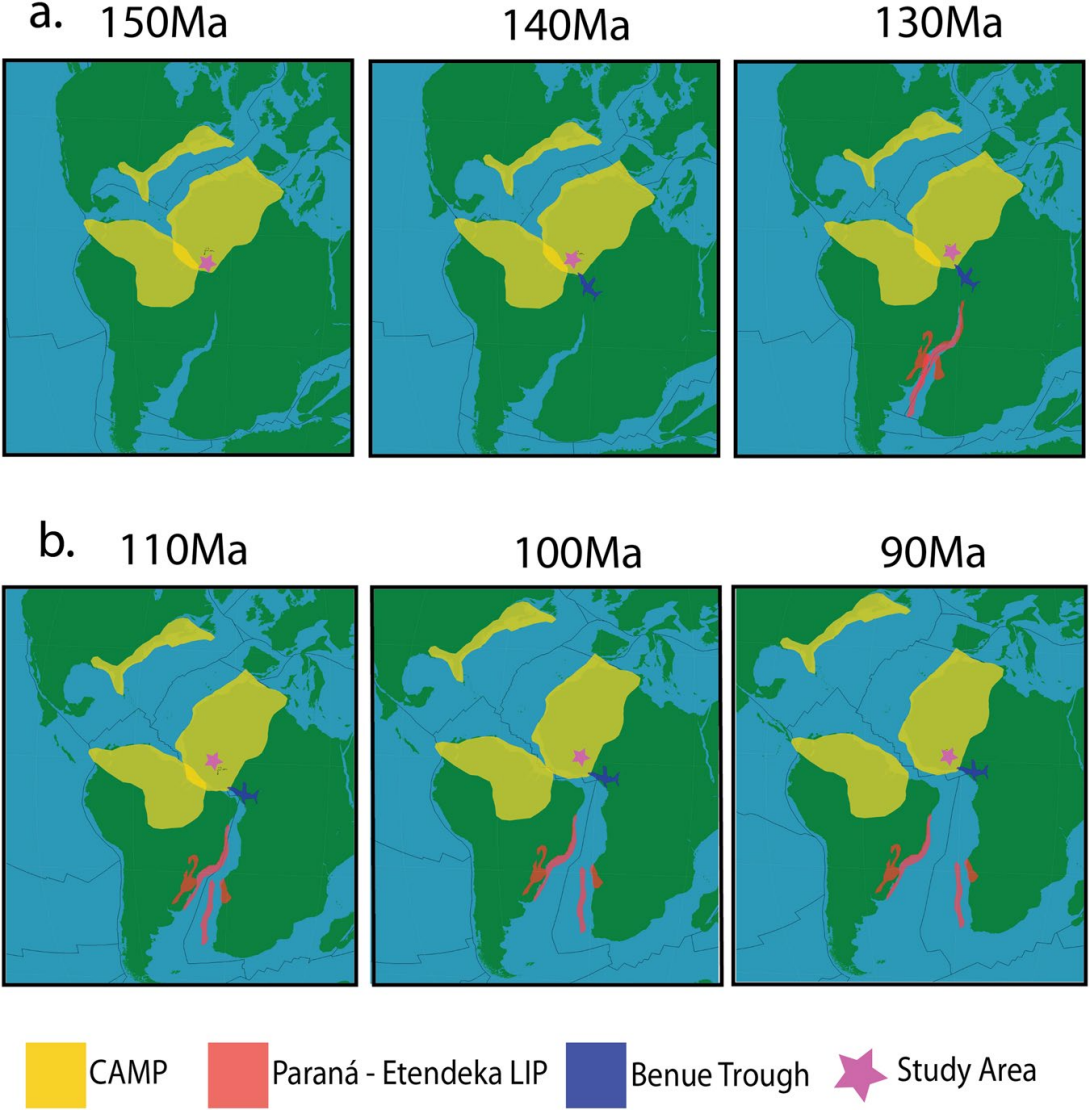
Time sequence map showing the evolution of Gondwana break-up in the Atlantic ocean. (**a)** The evolution of Gondawana break-up between 150 Ma and 130 Ma in context with the early Cretaceous AFT ages presented in this study. (Modified from plate reconstruction models by^61^). (**b**) The evolution of Gondawana break-up between 110 Ma and 90 Ma in context with the middle to Late Cretaceous AFT ages presented in this study. (Modified from^61^ utilizing plate rotation files available in the Gplates Software Package version 1.5.0. https://www.gplates.org/).

We propose that the Late Triassic – Early Jurassic cooling of the Ghanese upper crust is related to thermal relaxation after emplacement of the Central Atlantic Magmatic Province (CAMP) (Fig. 5a). Geochronological and ^40^Ar/^39^Ar studies that measure the emplacement ages for CAMP typically reveal a short duration for the most intense period of magmatism between 202 Ma and 189 Ma in the central and northern WAC, while in western Sierra Leone and towards the east, in Nigeria, manifestations of CAMP related volcanism span between ca. 234 Ma and ca. 140 Ma^15,18,19,21^. Although outcrop evidence for CAMP related volcanism is limited within the study area, the large extent of CAMP emplacement over four continents including Europe, South America, North America and Africa (Fig. 5) indicates that CAMP related heat flow likely also affected the study area in Ghana^14,15,20,36^. In addition, the Jurassic is the time when the extensive Karoo Large Igneous Province (LIP) and Basin in southern Africa formed^36^, which initiated the development of the so-called African erosion surface^37^. We suggest that the emplacement of LIPs such as CAMP caused a temporary increase in the geothermal gradient and the AFT ages in Ghana record the subsequent cooling (Fig. 3). Similar interpretations were made for other thermochronological studies conducted in NE Brazil^38,39^, juxtaposed to the Ghana margin and within the extent of CAMP. The study by^38^ reports AFT and Zircon Fission Track (ZFT) ages of approximately 200 Ma and interpret slow cooling related to the decline of magmatic activity in the region. The study by^39^ reports AFT ages of approximately 200 Ma and relate the data to crustal heating, induced by CAMP. Alternatively,^40^ describe substantial Permian – Jurassic plateau exhumation in NE Brazil to be related with erosion of topography that was generated during Gondwana amalgamation.

### Early to middle Cretaceous cooling

Off-shore studies conducted on the exhumed ICG margin indicate the onset of continental rifting and intracontinental deformation between the African and South American continents to be consistent with the younger observed (mid-Cretaceous) thermal event^6–8,16,23,41^. Extension in western Africa is generally regarded as occurring since the Late Permian and driven by far field tectonics during Pangea break-up with the aid of the emplacement of magmatic provinces (such as CAMP) through the Jurassic and Early Cretaceous (Fig. 5)^7,14,15,41^. In this context, Early to middle Cretaceous magmatism in the Benue Trough and related break-up in the South Atlantic is considered to be a major pre-cursor which drove the development of the equatorial transform zone during the latest Jurassic and earliest Cretaceous^15,17^. This event occurred due to a northward propagating rift axis and the emplacement of the St Helena plume head below the Benue Trough, creating a triple junction style break-up scenario which induced internal strain on the African plate (Fig. 5)^7,17,41^. As a result of continuous rifting along the African/South American margins prior to the final stages of rifting in the Equatorial Atlantic, this study assumes a passive rift model throughout the study area allowing for above average geothermal gradients^42^.

The thermal history models for samples NW of the BN shear zone reveal associated fast cooling during the Early and middle Cretaceous (Fig. 3), coinciding with the earliest stages of intra-continental break up, which resulted in rifting at the equatorial Atlantic during the middle Cretaceous^4,7–10,22,41,43^. The Early to middle Cretaceous cooling event is therefore interpreted in terms of exhumation in response to the two initial geodynamic stages of break-up. (1) The onset of rifting, intracontinental transform faulting and the initiation of the Deep Ivorian Basin during the Aptian and (2) the shift in the overall tectonic setting as a result of the change in drift orientation (from SW to SSW) of the South American plate with respect to the African plate from the Aptian to the Cenomanian^7,9,11,24^ (Fig. 5b). There are several examples of brittle shear zone reactivating during the early stages of rift evolution recorded in many areas around the globe. Examples of these include the North Atlantic rift, Rio Grande rift, East African rift and West European rift^44^. Furthermore,^44^ suggests that the brittle reactivation (due to rifting) of Neoproterozic shear zones in the Paraíba Basin in northeastern Brazil induced exhumation of the region, which indicates that a similar circumstance could occur on the opposite margin, within West Africa.

### Late Cretaceous – Cenozoic Cooling

Our thermochronological data and models provide minor evidence for a third cooling phase that affected the study area during the late Cretaceous – early Cenozoic. Late Cretaceous to early Cenozoic AFT ages (Table 2) were measured for samples located in the central BN shear zone and in vicinity to late brittle faults (D6 and D7 deformation phases) as recorded by^3^. Published off-shore AFT data from^7,9^ reveal cooling ages ranging between 65 Ma and 92 Ma, which are interpreted to constrain the final stages of break-up and exhumation of the ICG marginal ridge (Fig. 5b). We interpret the late Cretaceous – early Cenozoic cooling along the BN shear zone to be related with the exhuming ICG margin and/or with folding and stike-slip faulting as response to NNW-SSE shortening within western Africa^26,27^.

### Differential exhumation with respect to the Bole-Nangodi Shear Zone

**Figure 6:**
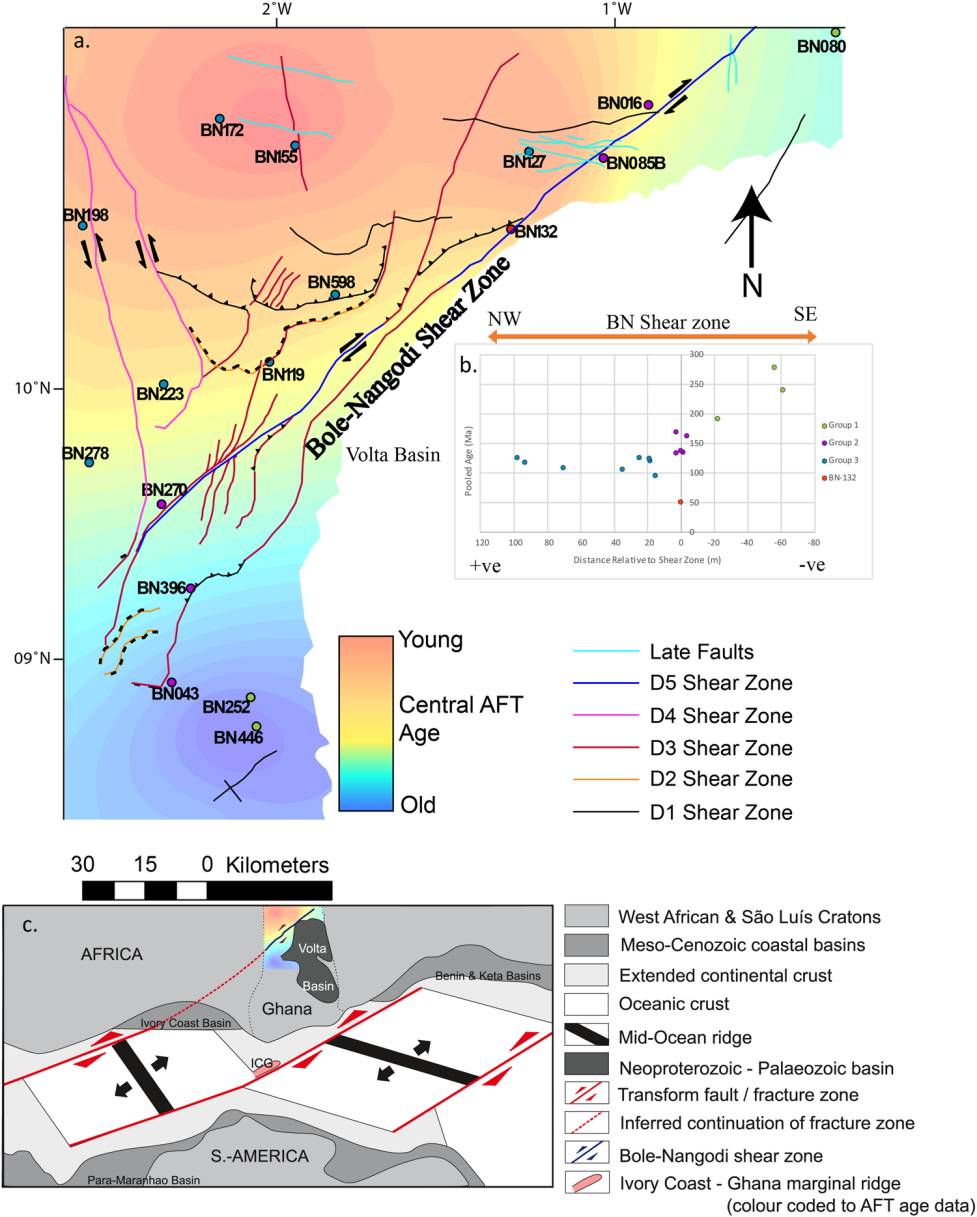
An Apatite Fission Track Map showing the differential exhumation throughout the study region. (**a**) Gridded ‘heat map’ for the study area defined by AFT pooled ages. Hot and cold colours represent young and old AFT ages, respectively. Faults are indicated and coloured according to relative deformation events (absolute timings of D4, D5 and ‘Late Fault’ (D6 and D7) activity are not known). Modified from^3^. (**b**) Scatter plot showing the relationship between pooled age (Ma) (Y-axis) and position (m) relative to the BN shear zone (X-axis) where -ve distances represent samples SE of the BN shear zone and +ve distances represent samples NW of the BN shear zone. Modified after^3^ utilizing ArcGIS Software Package version 10.3.1 http://desktop.arcgis.com/en/arcmap/. (**c**) Palaeogeographic reconstruction of the late Albian – early Cenomanian (~100 Ma), modified from^62^, illustrating our interpretation in which we suggest that the BN shear zone was reactivated in response to continental break up and transform fault development.

The BN shear zone acts as the most significant structure within the study area and is oriented parallel to the modern expression of transform zones in the equatorial Atlantic Ocean (Fig. 1). A gridded interpolation map (Fig. 6) was constructed to assess the role of the shear zone in relation to the cooling history of the study area. This map reveals distinctively different cooling ages for samples northwest of the BN shear zone compared to samples southeast of the BN shear zone, which is interpreted as a record of differential exhumation with respect to the shear zone. More specifically, our data suggests that the BN shear zone was reactivated during middle Cretaceous extension, exhuming the north-western block (footwall) with respect to the subsiding south-eastern block (hanging wall, comprising the Volta Basin). Therefore the north-western block exposes a deeper section of the northern Ghanese thermal history, compared to the south-eastern block. This record of differential exhumation supports the interpretation by^10^ that the BN shear zone acted as a control on the NE-SW orientation of the Atlantic transform fault that formed during Gondwana break-up (Fig. 6). Structural observations within the study area describe deformation phase D5 as localised E-W to NE-SW striking brittle deformation associated with dextral strike-slip shearing under a transcurrent regime^3^. Given the excellent correlation of the D5 fault trend with the significant step in AFT ages on the gridded AFT map (Fig. 6), this work dates the D5 brittle deformation phase to the early-middle Cretaceous. The D5 fault trend can be extrapolated to the St-Paul fracture zone, suggesting that strain may have partitioned towards the Bole-Nangodi shear zone during rifting and transform fault development (Fig. 6c).

The youngest AFT central ages measured in this study are located in the central section of the BN shear zone (Fig. 6) where there is a significant wedge-shaped geometry. This region is bound by faults of either D5 or ‘Late Fault’ relative ages. Therefore, the ‘Late Faults’ are interpreted to be related with the youngest AFT ages for this study (Late Cretaceous – Cenozoic). Given the similarities in AFT age data and structural setting, this work further relates the tectonic interpretation for the study area with those obtained for the ICG margin and the Gombe Fault in the Upper Benue Trough^4,23,24,27^. According to field studies, stratigraphic correlations and (for the case of the ICG margin) low temperature thermochronology, both the Gombe Fault and ICG margin were active and exhumed during the Late Cretaceous (towards the end of the Equatorial Atlantic rifting period) as the equatorial margin became passive^7,9,26,27^. Previous studies conducted by^45^ have noted similar styles of reactivation along pre-existing shear zones in the South Tien Shan (Central Asia). In light of these relationships, we suggest that the youngest region of the study area (Fig. 6), located in the central BN shear zone, is interpreted as having undergone minor Late Cretaceous to early Cenozoic exhumation in response to a compressional stress regime, as discussed above^26^. We, therefore, interpret the ‘Late Fault’ stage brittle faults located at the central BN shear zone near samples BN-127 and BN-016 to represent step-over faults or restraining bends. These structures are typically associated with strike-slip faults of this nature^46^. Further work would be required to confirm this interpretation, as structural field observations around brittle deformation zones within the study area are limited.

## Conclusions

Apatite fission track thermochronological results from northern Ghana characterise the thermal and deformation history of the NE-SW striking Bole-Nangodi shear zone and the surrounding area in context with continental rifting between West Africa and Brazil. Our thermal history models suggest that the Ghanese crust cooled during the Late Triassic - Early Jurassic, which post-dates the emplacement of CAMP and coincides with the development of the Karoo Basin and LIP. The cooling signal further corresponds to the time of the development of the African erosion surface. Samples to the northwest of the Bole-Nangodi shear zone record early to middle Cretaceous cooling, contemporaneous with the initiation of rifting in the equatorial Atlantic. Our data suggest that the shear zone was reactivated at that time, exhuming northwestern Ghana with respect to the southeast. Late Cretaceous – Cenozoic cooling was revealed for samples in the vicinity of late brittle faults and coincides with the timing of kilometre-scale exhumation in the Ivory Coast-Ghana margin and NNW-SSE shortening in western Africa. Our study furthermore suggests that strain associated with the St-Paul transform fault may have propagated into the continental interior to reactivate the Bole-Nangodi shear zone (Fig. 6c), demonstrating the role of inherited on-shore structures during Equatorial Atlantic rifting.

## Methods

### Apatite Fission Track thermochronology

Apatite Fission Track thermochronology is based on the spontaneous fission decay of ^238^Uranium and is used to constrain the low-temperature (~60–120 °C) thermal history through the apatite fission track partial annealing zone (APAZ)^47^. For this study, apatite grains were picked, mounted in epoxy resin, ground and polished to expose internal apatite sections. The apatite mounts were etched in 5 M HNO3 for 20.0 ± 0.5 s at 20.0° ± 0.5 °C to expose the spontaneous fission tracks and subsequently imaged on a Zeiss AXIO Imager M2m Autoscan System at The University of Adelaide. Fission track densities, Dpar and lengths were measured using the FastTracks software. Where possible, a minimum of 20 grains were analysed per sample. The objective was to measure at least 100 confined fission track lengths, a target that was not always achieved. The ^238^U and ^35^Cl concentration of each counted grain were measured using spot analyses on a New Wave UP213 laser connected to an Agilent-7500cs ICPMS^48^. U concentrations were determined by measuring ^238^U/^43^Ca ratios, calibrated against NIST610, NIST612 and Madagascar apatite standards (U-Pb age of 473.5 ± 0.7 Ma^49^). Age calculations were carried out following^50^ using a zeta approach^51^ with Durango as secondary age standard (^40^Ar/^39^Ar age of 31.44 ± 0.18 Ma^52^). A total of 102 measurements on Durango apatite were carried out for this study, spread over 3 different (well-characterised and homogeneous in U) crystals with mean U concentrations of 15.54 ± 0.08 ppm, 12.67 ± 0.07 ppm and 9.80 ± 0.10 ppm. The resulting mean AFT age of 31.65 ± 0.7 Ma for Durango, obtained in this study, is in excellent agreement with the published age. Accuracy of the applied methodology against the traditional external detector method is demonstrated in^53^ ^35^Cl measurements were carried out following^54^, who were able to demonstrate that electron probe and LA-ICP-MS data are the same when analysing a number of different apatites with different ^35^Cl compositions. Durango was used as accuracy check, producing a mean Cl concentration of 4.4 ± 0.1 ppm, which is in good agreement with published values. Data reduction was performed using the Trace_Element_IS data reduction scheme in the Iolite software^55^. More details on the methodology, can be found in^33,50^.

### Data Presentation and Modelling

AFT central ages were calculated with RadialPlotter^56^ and represent the apparent AFT cooling age of the analysed samples. This central age is not necessarily a good estimate of a cooling event and therefore subsequent thermal history modelling is needed to derive the tT history preserved in the sample. Furthermore, it is possible to (partially) preserve multiple AFT cooling ages or ‘peak ages’ in samples which fail Pearsons χ^2^ test or where single-grain ages show significant dispersion (above an arbitrary value of ~25%) which is regarded as a beyond natural spread^57^. In more detail, radial plots often show an open-jaw display of an older event and younger age component that can be related to distinct cooling events^58^. This is especially the case when two apatite populations have significantly different chemistries. In this regard, chlorine concentrations are often used as a chemical discriminator^30^. Elevated chlorine concentrations are known to slow fission track annealing but often show only little differences between apatite populations. This study uses uranium concentrations in addition to chlorine concentrations as a discriminator, which is thought to have a similar effect on fission track annealing. In this regard^34^, described a model of radiation-enhanced annealing for apatites with elevated uranium concentrations from old cratonic rocks. More recent observations^35^; confirm that alpha-particle induced annealing of radiation damage occurs in apatite and should be considered alongside with the better understood thermal annealing.

Thermal history (time-temperature) modelling was performed using the software package QTQt which uses the Bayesian trans-dimensional Markov Chain Monte Carlo statistical method to derive the most likely thermal history models^59^. The input parameters required in our modelling approach include individual AFT ages, their standard deviations, confined track lengths, sample elevations and Dpar used as a proxy for apatite chemistry as kinetic parameter^59^. The final output of each modelling attempt resolves three different models (Max. Likelihood, Max. Posterior and Max. Mode) and generates a weighted mean model or ‘Expected’ model based on a probability function at each point in the time-temperature space. The probability function is visualised in a colour spectrum ranging from blue (low probability) to red (high probability) where the absolute numerical probability value varies per model (Supplementary Fig. S5). Further details on the QTQt modelling approach and modelling outputs, can be found in^59^. For comparison purposes, a smooth spline was fitted through the ‘maximum mode’ for each model output (Supplementary Fig. S5). (Supplementary Fig. S5). These splines are the best representations of the most likely tT path and are compared to each other in Fig. 3. Model parameters are summarised in Supplementary Table 2, following the recommendations by^60^.

## Electronic supplementary material

Supplementary Information

## Data Availability

All data generated or analysed during this study are included with the initial submission of the article in the form of *Supplementary Information* and are available on request.
