# Supplementary material for: Thermochronological insights into reactivation of a continental shear zone in response to Equatorial Atlantic rifting (northern Ghana)
Source: Sci Rep. 2018 Nov 9;8:16619. doi: 10.1038/s41598-018-34769-x (PMC6226541; doi:10.1038/s41598-018-34769-x)
Supplement: Supplementary file 1 — Supplementary Information [file 41598_2018_34769_MOESM1_ESM.docx]

**Thermochronological insights into reactivation of a continental shear zone in response to Equatorial Atlantic rifting (northern Ghana).**

Nicholas Fernie^a^, Stijn Glorie^a,*^, Mark W. Jessell^b^, Alan S. Collins^a^

*^a^ Centre for Tectonics, Resources and Exploration (TRaX), Department of Earth Sciences, School of Physical Sciences, The University of Adelaide, Adelaide SA-5005, Australia*

*^b^ Centre for Exploration Targeting, School of Earth and Environment, The University of Western Australia, Crawley, WA 6009, Australia*

[nicholas.fernie@adelaide.edu.au](mailto:nicholas.fernie@adelaide.edu.au)

| Name | 238U Dur (ppm) | SD (ppm) | RF | Ns | Area (cm^2) | rho s | t (Ma) | SD t (Ma) |
| --- | --- | --- | --- | --- | --- | --- | --- | --- |
| 016_1.D | 14.3 | 0.68 | 4.75524476 | 86 | 5.63E-05 | 1528345.477 | 209.7763371 | 24.72 |
| 016_2.D | 9.49 | 0.6 | 6.32244468 | 30 | 2.62E-05 | 1147227.533 | 236.7770365 | 45.75 |
| 016_3.D | 11.06 | 0.65 | 5.87703436 | 69 | 7.08E-05 | 974713.9426 | 173.4690971 | 23.24 |
| 016_4.D | 29 | 1.1 | 3.79310345 | 142 | 9.28E-05 | 1530667.242 | 104.45183 | 9.62 |
| 016_5.D | 9.81 | 0.49 | 4.99490316 | 65 | 7.00E-05 | 928836.8105 | 186.1830825 | 24.9 |
| 016_6.D | 14.53 | 0.85 | 5.84996559 | 99 | 1.15E-04 | 862369.338 | 117.3345086 | 13.64 |
| 016_7.D | 14.8 | 1.2 | 8.10810811 | 148 | 1.18E-04 | 1255301.103 | 167.0336584 | 19.29 |
| 016_8.D | 18.9 | 1.2 | 6.34920635 | 58 | 3.83E-05 | 1513569.937 | 157.8229125 | 23.02 |
| 016_9.D | 22.8 | 2 | 8.77192982 | 50 | 4.31E-05 | 1161440.186 | 100.8364095 | 16.78 |
| 016_10.D | 11.35 | 0.89 | 7.84140969 | 69 | 4.81E-05 | 1433319.485 | 247.1450653 | 35.51 |
| 016_11.D | 13.08 | 0.78 | 5.96330275 | 56 | 3.99E-05 | 1405269.762 | 210.8562039 | 30.86 |
| 016_12.D | 16.79 | 0.94 | 5.59857058 | 45 | 4.15E-05 | 1083293.211 | 127.4534097 | 20.3 |
| 016_13.D | 14.25 | 0.89 | 6.24561404 | 35 | 3.07E-05 | 1139322.917 | 157.5686927 | 28.39 |
| 016_14.D | 14.8 | 1.4 | 9.45945946 | 61 | 5.96E-05 | 1023146.595 | 136.4669932 | 21.72 |
| 016_15.D | 11.32 | 0.85 | 7.50883392 | 79 | 6.63E-05 | 1191913.096 | 206.7153975 | 27.96 |
| 016_16.D | 11.41 | 0.67 | 5.87204207 | 78 | 4.78E-05 | 1631116.688 | 279.0752378 | 35.6 |
| 016_17.D | 19.7 | 1.1 | 5.58375635 | 33 | 4.10E-05 | 805270.8638 | 81.04002007 | 14.82 |
| 016_18.D | 20 | 1.3 | 6.5 | 107 | 6.96E-05 | 1538461.538 | 151.6680725 | 17.67 |
| 016_19.D | 47.1 | 3.8 | 8.06794055 | 108 | 3.43E-05 | 3152364.273 | 132.163771 | 16.6 |
| 016_20.D | 11.3 | 1.1 | 9.73451327 | 23 | 3.27E-05 | 703363.9144 | 123.0008865 | 28.3 |
| 016_21.D | 12.44 | 0.77 | 6.18971061 | 62 | 4.89E-05 | 1266857.376 | 200.0360104 | 28.26 |
| 016_22.D | 37.6 | 3.9 | 10.3723404 | 35 | 2.66E-05 | 1317275.122 | 69.51864168 | 13.79 |
| 016_23.D | 12.36 | 0.78 | 6.31067961 | 46 | 4.67E-05 | 985643.8826 | 157.164049 | 25.21 |
| 016_24.D | 16.57 | 0.88 | 5.31080266 | 63 | 6.67E-05 | 944669.3657 | 112.7483858 | 15.42 |
| 016_25.D | 17.7 | 1.1 | 6.21468927 | 75 | 5.69E-05 | 1317407.342 | 146.8076504 | 19.25 |
| 016_26.D | 22.1 | 2.4 | 10.8597285 | 98 | 6.61E-05 | 1483275.314 | 132.5299549 | 19.66 |
| 016_28.D | 9.47 | 0.79 | 8.34213305 | 64 | 6.57E-05 | 973828.3628 | 201.9612691 | 30.35 |
| 016_29.D | 30.8 | 2.1 | 6.81818182 | 104 | 4.37E-05 | 2378230.048 | 152.2374187 | 18.18 |
| 016_30.D | 19.7 | 1.4 | 7.10659898 | 91 | 6.19E-05 | 1471301.536 | 147.3060166 | 18.66 |
| 016_31.D | 32.8 | 2.5 | 7.62195122 | 43 | 2.55E-05 | 1688923.802 | 101.9189692 | 17.38 |
| 016_32.D | 13.7 | 1.2 | 8.75912409 | 35 | 3.23E-05 | 1084934.904 | 156.0885826 | 29.72 |
| 016_33.D | 10.74 | 0.72 | 6.70391061 | 40 | 3.64E-05 | 1099807.534 | 201.1297051 | 34.54 |
| 016_34.D | 21.3 | 2.6 | 12.2065728 | 67 | 3.43E-05 | 1955633.392 | 180.6200364 | 31.19 |
| 016_35.D | 24.4 | 1.8 | 7.37704918 | 25 | 1.44E-05 | 1733703.19 | 140.2198601 | 29.89 |
| 016_36.D | 13.9 | 1.3 | 9.35251799 | 50 | 4.69E-05 | 1065189.604 | 151.1015078 | 25.62 |
| 016_37.D | 20.4 | 1.6 | 7.84313725 | 52 | 5.53E-05 | 940495.5688 | 91.32781145 | 14.55 |
| 016_38.D | 27.8 | 3.7 | 13.3093525 | 36 | 6.03E-05 | 597411.2181 | 42.73117692 | 9.11 |
| 016_39.D | 4.09 | 0.3 | 7.33496333 | 24 | 5.36E-05 | 447928.3315 | 214.8738531 | 46.61 |
| 016_40.D | 15.49 | 0.91 | 5.87475791 | 49 | 4.81E-05 | 1018287.614 | 129.8358594 | 20.06 |
| 016_41.D | 19.6 | 1.5 | 7.65306122 | 52 | 3.77E-05 | 1378579.003 | 138.8185707 | 21.99 |
| 016_42.D | 21.6 | 1.9 | 8.7962963 | 46 | 3.32E-05 | 1387213.51 | 126.8718712 | 21.78 |
| 016_43.D | 10.62 | 0.66 | 6.21468927 | 33 | 4.50E-05 | 732844.7702 | 136.221994 | 25.18 |
| 016_44.D | 7.37 | 0.48 | 6.51289009 | 64 | 9.00E-05 | 711506.3924 | 189.7838456 | 26.75 |
| 016_45.D | 19.4 | 1.4 | 7.21649485 | 39 | 2.81E-05 | 1389878.831 | 141.3711838 | 24.83 |
| 016_46.D | 15.9 | 1.1 | 6.91823899 | 26 | 3.77E-05 | 690204.4067 | 86.02724725 | 17.89 |
| 016_47.D | 34.2 | 2.4 | 7.01754386 | 75 | 4.00E-05 | 1875000 | 108.4608525 | 14.66 |
| 016_48.D | 14 | 0.85 | 6.07142857 | 32 | 3.76E-05 | 852197.0706 | 120.3121418 | 22.49 |
| 016_49.D | 8.7 | 0.75 | 8.62068966 | 74 | 6.02E-05 | 1229440.106 | 275.9410878 | 39.94 |
| Name | 238U Dur (ppm) | SD (ppm) | RF | Ns | Area (cm^2) | rho s | t (Ma) | SD t (Ma) |
| 043_1.D | 26.1 | 2.2 | 8.42911877 | 53 | 2.73E-05 | 1941391.941 | 130.2976436 | 21 |
| 043_2.D | 26.4 | 2.2 | 8.33333333 | 31 | 2.36E-05 | 1315231.226 | 87.55998968 | 17.34 |
| 043_3.D | 25.5 | 1.9 | 7.45098039 | 47 | 2.60E-05 | 1808387.841 | 124.2849235 | 20.36 |
| 043_4.D | 28.3 | 2.3 | 8.12720848 | 33 | 1.64E-05 | 2017114.914 | 124.9079846 | 24 |
| 043_5.D | 33.2 | 3.2 | 9.63855422 | 25 | 1.66E-05 | 1505117.399 | 79.72678321 | 17.7 |
| 043_6.D | 37.8 | 2.7 | 7.14285714 | 29 | 1.58E-05 | 1834282.1 | 85.30177792 | 16.97 |
| 043_7.D | 22.4 | 1.4 | 6.25 | 33 | 1.98E-05 | 1670886.076 | 130.6623133 | 24.17 |
| 043_8.D | 25.5 | 1.7 | 6.66666667 | 16 | 1.43E-05 | 1117318.436 | 77.07230201 | 19.94 |
| 043_9.D | 30.9 | 2.4 | 7.76699029 | 20 | 1.50E-05 | 1329787.234 | 75.70618037 | 17.92 |
| 043_10.D | 34.2 | 3.2 | 9.35672515 | 34 | 1.83E-05 | 1854882.706 | 95.26594394 | 18.61 |
| 043_11.D | 30.7 | 2.8 | 9.12052117 | 41 | 2.78E-05 | 1475881.929 | 84.51302026 | 15.28 |
| 043_12.D | 45.8 | 3.7 | 8.07860262 | 47 | 2.60E-05 | 1808387.841 | 69.49337858 | 11.59 |
| 043_13.D | 29.7 | 2.8 | 9.42760943 | 32 | 1.70E-05 | 1882352.941 | 111.1870489 | 22.28 |
| 043_14.D | 34.8 | 3.4 | 9.77011494 | 38 | 2.06E-05 | 1843765.163 | 93.07811219 | 17.63 |
| 043_15.D | 38 | 2.8 | 7.36842105 | 96 | 2.92E-05 | 3287671.233 | 151.3067711 | 19.05 |
| 043_16.D | 37.1 | 2.8 | 7.54716981 | 53 | 2.00E-05 | 2652652.653 | 125.2966151 | 19.64 |
| 043_17.D | 36.1 | 3.2 | 8.86426593 | 43 | 1.41E-05 | 3060498.221 | 148.2996733 | 26.16 |
| 043_18.D | 25.3 | 3.2 | 12.6482213 | 34 | 1.24E-05 | 2741935.484 | 188.9799318 | 40.27 |
| 043_19.D | 31.7 | 3.2 | 10.0946372 | 57 | 2.59E-05 | 2198225.993 | 121.5548752 | 20.24 |
| 043_20.D | 26.7 | 2.8 | 10.4868914 | 50 | 1.63E-05 | 3069367.71 | 200.2784087 | 35.26 |
| 043_21.D | 21 | 1.3 | 6.19047619 | 30 | 2.16E-05 | 1391465.677 | 116.1966361 | 22.4 |
| 043_22.D | 29.2 | 3.1 | 10.6164384 | 111 | 3.41E-05 | 3253223.916 | 194.1930173 | 27.65 |
| 043_23.D | 43.6 | 2.5 | 5.73394495 | 82 | 3.03E-05 | 2702702.703 | 108.768486 | 13.53 |
| 043_24.D | 31.4 | 2.5 | 7.96178344 | 72 | 1.87E-05 | 3842049.093 | 212.9610786 | 30.29 |
| 043_25.D | 25.5 | 2.4 | 9.41176471 | 68 | 2.47E-05 | 2755267.423 | 188.4176518 | 28.92 |
| 043_26.D | 34.4 | 2 | 5.81395349 | 60 | 2.20E-05 | 2727272.727 | 138.7864394 | 19.65 |
| 043_27.D | 21.9 | 1.9 | 8.67579909 | 98 | 3.01E-05 | 3256895.979 | 257.9305203 | 34.35 |
| 043_28.D | 35.6 | 3.5 | 9.83146067 | 78 | 2.18E-05 | 3571428.571 | 175.1216979 | 26.26 |
| 043_29.D | 40.7 | 2.6 | 6.38820639 | 83 | 2.87E-05 | 2892994.075 | 124.5691728 | 15.82 |
| 043_30.D | 24.3 | 2 | 8.23045267 | 87 | 3.03E-05 | 2867501.648 | 205.5024394 | 27.78 |
| 043_31.D | 18.4 | 1.1 | 5.97826087 | 31 | 1.31E-05 | 2361005.331 | 223.1520611 | 42.24 |
| 043_32.D | 29.5 | 2.7 | 9.15254237 | 40 | 1.45E-05 | 2756719.504 | 163.2748739 | 29.83 |
| 043_33.D | 31.1 | 1.9 | 6.10932476 | 41 | 1.56E-05 | 2628205.128 | 147.8325033 | 24.79 |
| 043_34.D | 25.6 | 1.7 | 6.640625 | 26 | 1.48E-05 | 1761517.615 | 120.6251169 | 24.98 |
| 043_35.D | 26.6 | 2.2 | 8.27067669 | 27 | 1.52E-05 | 1779828.609 | 117.3271985 | 24.58 |
| 043_36.D | 31.4 | 2.1 | 6.68789809 | 30 | 1.38E-05 | 2170767.004 | 121.1867968 | 23.56 |
| 043_37.D | 21.1 | 1.8 | 8.53080569 | 22 | 1.51E-05 | 1458885.942 | 121.2020903 | 27.83 |
| 043_38.D | 29 | 2.2 | 7.5862069 | 26 | 1.35E-05 | 1920236.337 | 116.1180391 | 24.42 |
| 043_39.D | 44.2 | 3.9 | 8.82352941 | 73 | 2.32E-05 | 3150625.809 | 124.9166304 | 18.31 |
| 043_40.D | 29.9 | 3.4 | 11.3712375 | 42 | 1.16E-05 | 3626943.005 | 211.1532398 | 40.47 |
| 043_41.D | 31.6 | 3.3 | 10.443038 | 35 | 1.58E-05 | 2220812.183 | 123.1768924 | 24.47 |
| Name | 238U Dur (ppm) | SD (ppm) | RF | Ns | Area (cm^2) | rho s | t (Ma) | SD t (Ma) |
| 80_1.D | 4.2 | 0.31 | 7.38095238 | 18 | 3.49E-05 | 515759.3123 | 248.4030282 | 61.35 |
| 80_2.D | 4.62 | 0.35 | 7.57575758 | 16 | 4.20E-05 | 380771.0614 | 167.7690412 | 43.83 |
| 80_3.D | 5.01 | 0.42 | 8.38323353 | 14 | 2.71E-05 | 515843.773 | 208.9187666 | 58.52 |
| 80_4.D | 3.69 | 0.23 | 6.23306233 | 27 | 6.84E-05 | 394852.296 | 216.986164 | 43.89 |
| 80_5.D | 4.4 | 0.34 | 7.72727273 | 29 | 5.87E-05 | 494205.8623 | 227.5727712 | 45.77 |
| 80_6.D | 4.28 | 0.36 | 8.41121495 | 7 | 1.92E-05 | 365344.4676 | 173.6795384 | 67.25 |
| 80_7.D | 5.52 | 0.28 | 5.07246377 | 26 | 3.36E-05 | 773579.2919 | 282.7228159 | 57.27 |
| 80_8.D | 3.77 | 0.31 | 8.22281167 | 11 | 2.81E-05 | 390902.6297 | 210.3658405 | 65.74 |
| 80_9.D | 4.58 | 0.28 | 6.11353712 | 10 | 2.25E-05 | 445434.2984 | 197.5155527 | 63.62 |
| 80_10.D | 3.2 | 0.71 | 22.1875 | 12 | 2.58E-05 | 465296.6266 | 293.1046516 | 106.72 |
| 80_11.D | 5.65 | 0.39 | 6.90265487 | 13 | 2.85E-05 | 456300.4563 | 164.4390545 | 47 |
| 80_12.D | 3.61 | 0.31 | 8.58725762 | 8 | 3.37E-05 | 237247.9241 | 134.1289945 | 48.8 |
| 80_13.D | 4.39 | 0.21 | 4.78359909 | 15 | 4.17E-05 | 359971.2023 | 166.925103 | 43.83 |
| 80_14.D | 4.18 | 0.38 | 9.09090909 | 23 | 4.66E-05 | 493986.2543 | 239.2262046 | 54.42 |
| 80_15.D | 5.53 | 0.34 | 6.1482821 | 14 | 3.84E-05 | 365058.6701 | 134.7239707 | 36.95 |
| 80_18.D | 8.76 | 0.69 | 7.87671233 | 15 | 3.96E-05 | 378883.5565 | 88.5865145 | 23.91 |
| 80_19.D | 3.17 | 0.26 | 8.20189274 | 7 | 3.61E-05 | 193959.5456 | 124.9651922 | 48.33 |
| 80_20.D | 4 | 0.33 | 8.25 | 10 | 2.51E-05 | 397930.76 | 201.9667739 | 66.01 |
| 80_21.D | 5.88 | 0.5 | 8.50340136 | 16 | 3.26E-05 | 490346.3071 | 169.7263327 | 44.82 |
| 80_22.D | 4.78 | 0.32 | 6.69456067 | 19 | 3.89E-05 | 488055.4842 | 207.2031666 | 49.52 |
| 80_23.D | 3.77 | 0.26 | 6.89655172 | 17 | 3.39E-05 | 501474.9263 | 268.6460916 | 67.74 |
| 80_24.D | 4.93 | 0.25 | 5.07099391 | 12 | 3.01E-05 | 399334.4426 | 164.9210387 | 48.34 |
| 80_25.D | 4.22 | 0.28 | 6.63507109 | 24 | 3.80E-05 | 631911.5324 | 301.6454013 | 64.74 |
| 80_26.D | 4.07 | 0.28 | 6.87960688 | 18 | 4.07E-05 | 441934.6919 | 220.1308444 | 54.05 |
| 80_27.D | 3.44 | 0.19 | 5.52325581 | 26 | 4.27E-05 | 608329.4338 | 354.75545 | 72.28 |
| 80_28.D | 4.8 | 0.28 | 5.83333333 | 17 | 4.80E-05 | 354166.6667 | 150.3986157 | 37.52 |
| 80_29.D | 3.58 | 0.26 | 7.26256983 | 11 | 4.07E-05 | 270137.5246 | 153.7677802 | 47.69 |
| 80_30.D | 4.21 | 0.27 | 6.41330166 | 12 | 2.53E-05 | 473746.5456 | 227.9895392 | 67.42 |
| 80_31.D | 4.34 | 0.31 | 7.14285714 | 14 | 4.18E-05 | 335329.3413 | 157.4063413 | 43.55 |
| 80_32.D | 4.78 | 0.41 | 8.57740586 | 9 | 2.06E-05 | 436681.2227 | 185.7032949 | 63.92 |
| 80_34.D | 4.82 | 0.44 | 9.12863071 | 10 | 3.01E-05 | 332557.3661 | 140.7419085 | 46.32 |
| 80_35.D | 3.57 | 0.24 | 6.72268908 | 14 | 2.53E-05 | 553797.4684 | 312.2311427 | 86.05 |
| 80_37.D | 5.07 | 0.25 | 4.93096647 | 16 | 5.65E-05 | 283085.6334 | 114.1334722 | 29.08 |
| 80_39.D | 4.03 | 0.34 | 8.43672457 | 19 | 4.13E-05 | 459714.4931 | 231.0627692 | 56.48 |
| 80_40.D | 4.64 | 0.42 | 9.05172414 | 13 | 3.00E-05 | 432900.4329 | 189.5925995 | 55.31 |
| 80_41.D | 7.81 | 0.46 | 5.88988476 | 6 | 1.70E-05 | 353148.9111 | 92.58440009 | 38.19 |
| 80_42.D | 4.5 | 0.31 | 6.88888889 | 17 | 2.88E-05 | 590893.2916 | 265.2673493 | 66.88 |
| 80_43.D | 3.65 | 0.31 | 8.49315068 | 12 | 2.46E-05 | 487804.878 | 269.8881389 | 81.21 |
| 80_45.D | 3.84 | 0.28 | 7.29166667 | 12 | 2.88E-05 | 416377.5156 | 219.8282535 | 65.45 |
| 80_46.D | 4.38 | 0.32 | 7.30593607 | 8 | 2.39E-05 | 334308.3995 | 155.5168605 | 56.15 |
| 80_47.D | 4.02 | 0.34 | 8.45771144 | 11 | 2.43E-05 | 452674.8971 | 228.1424689 | 71.44 |
| Name | 238U Dur (ppm) | SD (ppm) | RF | Ns | Area (cm^2) | rho s | t (Ma) | SD t (Ma) |
| 85_1.D | 3.91 | 0.41 | 10.4859335 | 14 | 3.35E-05 | 418410.0418 | 207.0657564 | 59.45 |
| 85_2.D | 4.57 | 0.23 | 5.03282276 | 28 | 5.25E-05 | 533536.5854 | 225.5816408 | 44.12 |
| 85_3.D | 22.5 | 1.1 | 4.88888889 | 39 | 3.51E-05 | 1111427.757 | 96.41036082 | 16.14 |
| 85_5.D | 3.14 | 0.35 | 11.1464968 | 5 | 1.88E-05 | 266666.6667 | 164.8732176 | 75.99 |
| 85_6.D | 5.45 | 0.43 | 7.88990826 | 20 | 4.91E-05 | 407747.1967 | 145.4661219 | 34.49 |
| 85_7.D | 33.7 | 2 | 5.9347181 | 92 | 2.90E-05 | 3171320.234 | 182.4428042 | 21.89 |
| 85_8.D | 12.7 | 1.1 | 8.66141732 | 31 | 2.62E-05 | 1181402.439 | 180.3767693 | 35.97 |
| 85_9.D | 3.55 | 0.32 | 9.01408451 | 23 | 4.61E-05 | 499023.6494 | 270.6577053 | 61.48 |
| 85_11.D | 5.55 | 0.34 | 6.12612613 | 32 | 4.25E-05 | 752233.1923 | 261.161532 | 48.86 |
| 85_12.D | 10.05 | 0.69 | 6.86567164 | 14 | 3.50E-05 | 399771.5591 | 77.7500731 | 21.45 |
| 85_13.D | 6.84 | 0.6 | 8.77192982 | 31 | 3.45E-05 | 899071.9258 | 253.4255242 | 50.66 |
| 85_14.D | 7.8 | 1 | 12.8205128 | 12 | 2.73E-05 | 440044.0044 | 109.9936765 | 34.74 |
| 85_15.D | 17.3 | 1.2 | 6.93641618 | 38 | 3.85E-05 | 988296.4889 | 111.3681311 | 19.65 |
| 85_16.D | 5.82 | 0.64 | 10.9965636 | 11 | 2.85E-05 | 386235.9551 | 129.1954202 | 41.46 |
| 85_17.D | 3.46 | 0.23 | 6.64739884 | 17 | 4.41E-05 | 385574.9603 | 215.4915732 | 54.19 |
| 85_18.D | 3.69 | 0.36 | 9.75609756 | 11 | 3.44E-05 | 319953.4613 | 168.2890727 | 53.33 |
| 85_19.D | 5.26 | 0.38 | 7.2243346 | 22 | 2.49E-05 | 884599.9196 | 322.4995345 | 72.6 |
| 85_20.D | 5.03 | 0.44 | 8.74751491 | 18 | 2.64E-05 | 682076.5441 | 261.2827374 | 65.69 |
| 85_21.D | 4.09 | 0.3 | 7.33496333 | 30 | 3.69E-05 | 812567.714 | 379.2920895 | 74.63 |
| 85_22.D | 8.85 | 0.5 | 5.64971751 | 39 | 4.52E-05 | 862068.9655 | 188.7563579 | 32.05 |
| 85_23.D | 5.9 | 0.73 | 12.3728814 | 4 | 2.69E-05 | 148754.1837 | 49.38886749 | 25.44 |
| 85_24.D | 8.25 | 0.78 | 9.45454545 | 15 | 3.62E-05 | 413907.2848 | 97.90919818 | 26.92 |
| 85_25.D | 14.1 | 1.5 | 10.6382979 | 65 | 5.45E-05 | 1192004.401 | 164.1325926 | 26.82 |
| 85_26.D | 4.65 | 0.35 | 7.52688172 | 12 | 4.46E-05 | 268997.9825 | 112.7635427 | 33.64 |
| 85_27.D | 3.97 | 0.4 | 10.0755668 | 18 | 4.76E-05 | 377992.4402 | 184.5599627 | 47.31 |
| 85_28.D | 3.73 | 0.3 | 8.04289544 | 14 | 2.82E-05 | 496277.9156 | 256.4632469 | 71.58 |
| 85_29.D | 19.43 | 0.86 | 4.42614514 | 33 | 2.91E-05 | 1134410.45 | 113.7981253 | 20.44 |
| 85_30.D | 3.47 | 0.21 | 6.0518732 | 16 | 4.08E-05 | 391964.7232 | 218.3821613 | 56.17 |
| 85_31.D | 3.83 | 0.25 | 6.52741514 | 7 | 2.37E-05 | 295608.1081 | 150.0137868 | 57.54 |
| 85_32.D | 5.08 | 0.32 | 6.2992126 | 16 | 2.63E-05 | 608596.4245 | 231.3795513 | 59.65 |
| 85_33.D | 5.13 | 0.25 | 4.87329435 | 18 | 2.51E-05 | 715990.4535 | 268.7704404 | 64.69 |
| 85_34.D | 3.7 | 0.27 | 7.2972973 | 9 | 3.89E-05 | 231481.4815 | 121.8651851 | 41.58 |
| 85_35.D | 10.05 | 0.94 | 9.35323383 | 19 | 3.51E-05 | 541773.5957 | 105.1433533 | 26.05 |
| 85_36.D | 10.27 | 0.6 | 5.84225901 | 10 | 2.00E-05 | 501002.004 | 95.22131161 | 30.62 |
| 85_38.D | 3.55 | 0.4 | 11.2676056 | 15 | 2.76E-05 | 543675.2447 | 294.3307099 | 82.92 |
| 85_39.D | 7.67 | 0.87 | 11.3428944 | 36 | 3.88E-05 | 927595.9804 | 233.5338192 | 47.08 |
| 85_40.D | 14.9 | 2.8 | 18.7919463 | 23 | 2.50E-05 | 921843.6874 | 120.5261785 | 33.83 |
| 85_41.D | 3.05 | 0.27 | 8.85245902 | 9 | 2.14E-05 | 420954.1628 | 265.8434009 | 91.69 |
| 85_42.D | 3.61 | 0.37 | 10.2493075 | 4 | 2.08E-05 | 192678.2274 | 104.1095118 | 53.14 |
| 85_44.D | 4.31 | 0.35 | 8.12064965 | 16 | 3.87E-05 | 413223.1405 | 185.827232 | 48.85 |
| Name | 238U Dur (ppm) | SD (ppm) | RF | Ns | Area (cm^2) | rho s | t (Ma) | SD t (Ma) |
| 119_1.D | 4.37 | 0.29 | 6.63615561 | 10 | 2.46E-05 | 406669.3778 | 162.6041098 | 52.54 |
| 119_2.D | 7.4 | 1.2 | 16.2162162 | 8 | 1.20E-05 | 664451.8272 | 156.96185 | 61.05 |
| 119_3.D | 3.6 | 0.27 | 7.5 | 6 | 1.06E-05 | 566572.238 | 272.6444816 | 113.17 |
| 119_4.D | 3.64 | 0.39 | 10.7142857 | 8 | 2.50E-05 | 320384.4614 | 153.8990623 | 56.86 |
| 119_5.D | 9.2 | 2.5 | 27.173913 | 7 | 1.38E-05 | 507614.2132 | 96.90317092 | 45.11 |
| 119_6.D | 5.63 | 0.42 | 7.46003552 | 2 | 1.04E-05 | 193236.715 | 60.45102651 | 42.98 |
| 119_7.D | 3.25 | 0.21 | 6.46153846 | 8 | 2.08E-05 | 385542.1687 | 206.5722011 | 74.24 |
| 119_8.D | 2.62 | 0.26 | 9.92366412 | 2 | 1.51E-05 | 132362.6737 | 88.78313206 | 63.39 |
| 119_9.D | 4.39 | 0.47 | 10.7061503 | 15 | 2.08E-05 | 722891.5663 | 284.9926208 | 79.66 |
| 119_10.D | 6.57 | 0.46 | 7.00152207 | 3 | 1.02E-05 | 294117.6471 | 78.73379405 | 45.79 |
| 119_11.D | 6.55 | 0.69 | 10.5343511 | 14 | 2.03E-05 | 688298.9184 | 183.3185384 | 52.66 |
| 119_12.D | 4.09 | 0.3 | 7.33496333 | 6 | 1.44E-05 | 417827.2981 | 178.2848555 | 73.95 |
| 119_13.D | 4.1 | 0.37 | 9.02439024 | 1 | 1.31E-05 | 76394.19404 | 32.88732335 | 33.02 |
| 119_14.D | 3.64 | 0.27 | 7.41758242 | 4 | 1.38E-05 | 289226.3196 | 139.0922703 | 70.31 |
| 119_15.D | 3.43 | 0.27 | 7.87172012 | 6 | 1.16E-05 | 518134.715 | 261.9126606 | 108.89 |
| 119_16.D | 3.56 | 0.35 | 9.83146067 | 12 | 2.57E-05 | 467471.7569 | 228.2725277 | 69.61 |
| 119_17.D | 3.86 | 0.36 | 9.32642487 | 1 | 2.13E-05 | 46970.40864 | 21.4967609 | 21.59 |
| 119_18.D | 6.61 | 0.62 | 9.37972769 | 5 | 1.61E-05 | 310752.0199 | 82.65811571 | 37.77 |
| 119_19.D | 4.49 | 0.41 | 9.13140312 | 6 | 1.37E-05 | 437317.7843 | 170.0862331 | 71.15 |
| 119_20.D | 4.16 | 0.48 | 11.5384615 | 3 | 1.41E-05 | 212615.1665 | 89.81159483 | 52.88 |
| 119_21.D | 4.75 | 0.62 | 13.0526316 | 2 | 1.76E-05 | 113960.114 | 42.31490988 | 30.43 |
| 119_22.D | 5.85 | 0.64 | 10.9401709 | 4 | 1.13E-05 | 355555.5556 | 106.663055 | 54.59 |
| 119_24.D | 10.4 | 1 | 9.61538462 | 9 | 1.72E-05 | 522648.0836 | 88.31976182 | 30.64 |
| 119_25.D | 20.9 | 2.1 | 10.0478469 | 9 | 1.25E-05 | 718276.1373 | 60.52920137 | 21.07 |
| 119_26.D | 6.77 | 0.73 | 10.7828656 | 1 | 1.12E-05 | 89285.71429 | 23.29532004 | 23.43 |
| 119_27.D | 3.43 | 0.28 | 8.16326531 | 2 | 1.38E-05 | 144508.6705 | 74.12433807 | 52.76 |
| 119_28.D | 7.19 | 0.69 | 9.59666203 | 5 | 1.06E-05 | 470809.7928 | 114.8425161 | 52.53 |
| 119_29.D | 5.43 | 0.51 | 9.39226519 | 9 | 1.08E-05 | 834879.4063 | 266.487405 | 92.29 |
| 119_31.D | 6.92 | 0.9 | 13.0057803 | 5 | 1.55E-05 | 322372.6628 | 81.91251886 | 38.15 |
| 119_32.D | 4.13 | 0.35 | 8.47457627 | 3 | 7.93E-06 | 378548.8959 | 160.1862379 | 93.47 |
| 119_33.D | 3.04 | 0.25 | 8.22368421 | 2 | 7.80E-06 | 256508.9137 | 147.6072053 | 105.08 |
| 119_34.D | 4.17 | 0.43 | 10.3117506 | 4 | 1.41E-05 | 282885.4314 | 118.9384407 | 60.72 |
| 119_36.D | 3.66 | 0.41 | 11.2021858 | 12 | 1.20E-05 | 1000000 | 466.2086865 | 144.36 |
| 119_37.D | 3.17 | 0.3 | 9.4637224 | 6 | 2.39E-05 | 251151.1093 | 138.6934303 | 58.12 |
| Name | 238U Dur (ppm) | SD (ppm) | RF | Ns | Area (cm^2) | rho s | t (Ma) | SD t (Ma) |
| 127_1.D | 8.67 | 0.56 | 6.45905421 | 9 | 1.28E-05 | 704776.8207 | 157.8990255 | 53.61 |
| 127_2.D | 21 | 1.5 | 7.14285714 | 18 | 1.62E-05 | 1108374.384 | 102.9605753 | 25.36 |
| 127_4.D | 33.4 | 2.8 | 8.38323353 | 25 | 1.58E-05 | 1578282.828 | 92.25790083 | 20.01 |
| 127_5.D | 17.9 | 1.7 | 9.4972067 | 15 | 1.08E-05 | 1394052.045 | 151.3541167 | 41.64 |
| 127_6.D | 7.63 | 0.69 | 9.04325033 | 19 | 2.12E-05 | 897496.4573 | 227.2518159 | 56.04 |
| 127_7.D | 12.15 | 0.88 | 7.24279835 | 25 | 3.39E-05 | 737028.3019 | 118.1944388 | 25.14 |
| 127_9.D | 22.6 | 2.2 | 9.73451327 | 19 | 2.00E-05 | 950475.2376 | 82.1746742 | 20.48 |
| 127_10.D | 12.5 | 1.6 | 12.8 | 21 | 2.49E-05 | 842696.6292 | 131.2230466 | 33.2 |
| 127_11.D | 16.1 | 1.5 | 9.31677019 | 34 | 2.64E-05 | 1289833.08 | 155.6434529 | 30.38 |
| 127_12.D | 17.3 | 1 | 5.78034682 | 39 | 4.38E-05 | 889598.5401 | 100.3322385 | 17.08 |
| 127_13.D | 18.1 | 1.2 | 6.62983425 | 23 | 1.90E-05 | 1211801.897 | 130.3263502 | 28.52 |
| 127_14.D | 15.6 | 1.5 | 9.61538462 | 5 | 1.46E-05 | 343406.5934 | 43.14262612 | 19.73 |
| 127_15.D | 22.2 | 1.9 | 8.55855856 | 32 | 2.65E-05 | 1208459.215 | 106.1633517 | 20.85 |
| 127_16.D | 21.6 | 2 | 9.25925926 | 7 | 1.24E-05 | 566801.6194 | 51.39508093 | 20 |
| 127_19.D | 11.3 | 1 | 8.84955752 | 16 | 1.42E-05 | 1126760.563 | 193.1555211 | 51.22 |
| 127_20.D | 22.8 | 2 | 8.77192982 | 29 | 2.28E-05 | 1269702.277 | 108.5877308 | 22.3 |
| 127_21.D | 5.55 | 0.44 | 7.92792793 | 15 | 3.15E-05 | 476190.4762 | 166.5490119 | 44.98 |
| 127_22.D | 29.4 | 3.1 | 10.5442177 | 18 | 2.30E-05 | 783289.8172 | 52.17866255 | 13.47 |
| 127_23.D | 15 | 1 | 6.66666667 | 14 | 1.71E-05 | 816802.8005 | 106.198982 | 29.25 |
| 127_24.D | 14.6 | 0.85 | 5.82191781 | 11 | 1.50E-05 | 732356.8575 | 97.89147395 | 30.06 |
| 127_25.D | 27.7 | 2.1 | 7.58122744 | 19 | 1.82E-05 | 1043956.044 | 73.68761993 | 17.8 |
| 127_26.D | 17.9 | 1.7 | 9.4972067 | 10 | 1.15E-05 | 871839.5815 | 95.07223964 | 31.39 |
| 127_27.D | 27.3 | 2.1 | 7.69230769 | 13 | 2.28E-05 | 570175.4386 | 40.93951534 | 11.78 |
| 127_28.D | 23.7 | 2.7 | 11.3924051 | 13 | 1.78E-05 | 729108.2445 | 60.21302216 | 18.05 |
| 127_29.D | 13.92 | 0.85 | 6.10632184 | 13 | 2.05E-05 | 634146.3415 | 88.96651732 | 25.27 |
| 127_30.D | 18.9 | 2.6 | 13.7566138 | 6 | 9.05E-06 | 663056.6913 | 68.62019587 | 29.56 |
| Name | 238U Dur (ppm) | SD (ppm) | RF | Ns | Area (cm^2) | rho s | t (Ma) | SD t (Ma) |
| 132_1.D | 6.64 | 0.4 | 6.02409639 | 6 | 1.93E-05 | 311364.8158 | 91.55674305 | 37.78 |
| 132_2.D | 5.57 | 0.56 | 10.05386 | 6 | 3.02E-05 | 198741.3051 | 69.78414467 | 29.34 |
| 132_3.D | 4.43 | 0.68 | 15.3498871 | 4 | 2.03E-05 | 196947.3166 | 86.83496631 | 45.42 |
| 132_5.D | 4.43 | 0.41 | 9.25507901 | 12 | 5.28E-05 | 227401.9329 | 100.1587345 | 30.36 |
| 132_6.D | 10.51 | 0.96 | 9.13415794 | 3 | 1.48E-05 | 202292.65 | 37.73828582 | 22.06 |
| 132_8.D | 6.69 | 0.59 | 8.81913303 | 7 | 3.27E-05 | 213805.7422 | 62.54051936 | 24.27 |
| 132_9.D | 16.9 | 1.5 | 8.87573964 | 6 | 1.74E-05 | 344431.6877 | 39.95273441 | 16.69 |
| 132_10.D | 4.34 | 0.56 | 12.9032258 | 5 | 1.53E-05 | 325945.2412 | 146.017082 | 67.96 |
| 132_11.D | 13.54 | 0.91 | 6.72082718 | 4 | 1.70E-05 | 234741.784 | 34.00184606 | 17.15 |
| 132_12.D | 4.83 | 0.49 | 10.1449275 | 12 | 3.49E-05 | 344135.3599 | 138.6058258 | 42.41 |
| 132_13.D | 9.7 | 1.2 | 12.371134 | 2 | 2.01E-05 | 99750.62344 | 20.19018118 | 14.49 |
| 132_14.D | 22 | 1.9 | 8.63636364 | 3 | 1.52E-05 | 197498.3542 | 17.62882851 | 10.29 |
| 132_15.D | 3.81 | 0.43 | 11.2860892 | 2 | 2.10E-05 | 95374.3443 | 49.03772604 | 35.11 |
| Name | 238U Dur (ppm) | SD (ppm) | RF | Ns | Area (cm^2) | rho s | t (Ma) | SD t (Ma) |
| 155_01.D | 32.2 | 1.8 | 5.59006211 | 54 | 3.77E-05 | 1430842.607 | 102.2769885 | 15.05 |
| 155_02.D | 24 | 1.7 | 7.08333333 | 43 | 3.18E-05 | 1352201.258 | 129.4063078 | 21.76 |
| 155_03.D | 17.51 | 0.76 | 4.34037693 | 39 | 4.41E-05 | 884955.7522 | 116.2002389 | 19.28 |
| 155_04.D | 32.9 | 2 | 6.07902736 | 62 | 3.23E-05 | 1918910.554 | 133.9157837 | 18.86 |
| 155_05.D | 29.9 | 2 | 6.68896321 | 43 | 3.76E-05 | 1144225.652 | 88.17765314 | 14.68 |
| 155_06.D | 26.7 | 1.4 | 5.24344569 | 89 | 5.14E-05 | 1732866.044 | 148.8408278 | 17.6 |
| 155_07.D | 37.8 | 2.4 | 6.34920635 | 41 | 2.75E-05 | 1489825.581 | 90.79736031 | 15.31 |
| 155_08.D | 47.6 | 4.6 | 9.66386555 | 52 | 3.07E-05 | 1696574.225 | 82.16501482 | 13.89 |
| 155_09.D | 29 | 2.2 | 7.5862069 | 54 | 3.10E-05 | 1744749.596 | 138.0914817 | 21.51 |
| 155_10.D | 38.9 | 2.7 | 6.94087404 | 50 | 3.07E-05 | 1629195.178 | 96.44118496 | 15.19 |
| 155_11.D | 22 | 1.7 | 7.72727273 | 57 | 3.33E-05 | 1713770.295 | 178.2394356 | 27.33 |
| 155_13.D | 29.4 | 1.6 | 5.44217687 | 48 | 3.83E-05 | 1253591.016 | 98.17233113 | 15.14 |
| 155_14.D | 27 | 1.7 | 6.2962963 | 51 | 3.30E-05 | 1545923.007 | 131.4858934 | 20.19 |
| 155_15.D | 30.1 | 2 | 6.64451827 | 89 | 5.19E-05 | 1714175.655 | 130.7878754 | 16.36 |
| 155_16.D | 25 | 1.8 | 7.2 | 37 | 2.90E-05 | 1277183.293 | 117.4472088 | 21.08 |
| 155_17.D | 28.8 | 1.3 | 4.51388889 | 86 | 5.30E-05 | 1623254.058 | 129.4546716 | 15.13 |
| 155_18.D | 26.3 | 1.9 | 7.2243346 | 68 | 3.49E-05 | 1948424.069 | 169.6262438 | 23.94 |
| 155_19.D | 38.1 | 2.3 | 6.03674541 | 46 | 2.43E-05 | 1891447.368 | 114.1589229 | 18.19 |
| 155_20.D | 35.2 | 3.1 | 8.80681818 | 29 | 3.49E-05 | 829994.2759 | 54.47382659 | 11.2 |
| 155_21.D | 47.2 | 3.8 | 8.05084746 | 33 | 3.00E-05 | 1101836.394 | 53.93228639 | 10.34 |
| 155_22.D | 34.3 | 2.5 | 7.28862974 | 60 | 4.64E-05 | 1293382.194 | 86.89486457 | 12.88 |
| 155_23.D | 29.8 | 1.9 | 6.37583893 | 45 | 2.52E-05 | 1787132.645 | 137.6534537 | 22.32 |
| 155_24.D | 16.86 | 0.88 | 5.21945433 | 53 | 3.98E-05 | 1331992.963 | 180.7315167 | 26.56 |
| 155_27.D | 26.5 | 2.2 | 8.30188679 | 25 | 1.84E-05 | 1360174.102 | 117.9939216 | 25.55 |
| 155_28.D | 16.01 | 0.79 | 4.93441599 | 34 | 2.44E-05 | 1395157.981 | 199.067747 | 35.52 |
| 155_30.D | 44.3 | 3.3 | 7.44920993 | 66 | 2.86E-05 | 2307692.308 | 119.7364305 | 17.23 |
| 155_31.D | 29.7 | 1.5 | 5.05050505 | 42 | 2.95E-05 | 1423728.814 | 110.266326 | 17.9 |
| 155_32.D | 36.8 | 2.7 | 7.33695652 | 43 | 1.82E-05 | 2361339.923 | 147.1754426 | 24.91 |
| 155_33.D | 36.3 | 2.5 | 6.88705234 | 25 | 1.70E-05 | 1468860.165 | 93.20136052 | 19.71 |
| 155_36.D | 35.6 | 2.9 | 8.14606742 | 34 | 2.30E-05 | 1476335.215 | 95.50052719 | 18.13 |
| 155_37.D | 19.5 | 1.8 | 9.23076923 | 27 | 2.18E-05 | 1236830.05 | 145.4979243 | 31.06 |
| 155_38.D | 30.1 | 2 | 6.64451827 | 32 | 2.59E-05 | 1235044.384 | 94.49758823 | 17.85 |
| 155_39.D | 39.1 | 1.7 | 4.34782609 | 59 | 3.17E-05 | 1861786.052 | 109.5342214 | 15.03 |
| 155_40.D | 30 | 2.3 | 7.66666667 | 33 | 2.38E-05 | 1387720.774 | 106.4344821 | 20.25 |
| 155_41.D | 38.1 | 3.7 | 9.71128609 | 26 | 1.94E-05 | 1339515.714 | 81.05528235 | 17.74 |
| 155_42.D | 40.9 | 2.1 | 5.13447433 | 48 | 2.59E-05 | 1852566.577 | 104.2380364 | 15.97 |
| 155_45.D | 43.7 | 2.5 | 5.7208238 | 54 | 2.57E-05 | 2105263.158 | 110.8099271 | 16.36 |
| 155_46.D | 36.2 | 2.6 | 7.18232044 | 37 | 2.11E-05 | 1756885.09 | 111.6248716 | 20.03 |
| 155_47.D | 49.6 | 3.3 | 6.65322581 | 42 | 2.59E-05 | 1622874.807 | 75.46585358 | 12.68 |
| 155_48.D | 31.3 | 2.6 | 8.30670927 | 55 | 3.09E-05 | 1782242.385 | 130.7680829 | 20.71 |
| 155_50.D | 29.2 | 2.4 | 8.21917808 | 41 | 2.69E-05 | 1522465.652 | 119.8431394 | 21.15 |
| Name | 238U Dur | SD | RF | Ns | A (unadjusted) | rho s | t (Ma) | SD t |
| 172_1.D | 28.5 | 1.7 | 5.96491228 | 65 | 3.69E-05 | 1760563.38 | 108.3953632 | 14.92 |
| 172_2.D | 7.35 | 0.52 | 7.07482993 | 8 | 1.93E-05 | 415153.0877 | 99.18271543 | 35.76 |
| 172_3.D | 11.5 | 1.2 | 10.4347826 | 32 | 2.18E-05 | 1467216.873 | 221.9016806 | 45.55 |
| 172_4.D | 13.1 | 1 | 7.63358779 | 21 | 4.11E-05 | 510824.6169 | 68.63518492 | 15.87 |
| 172_5.D | 15.2 | 1 | 6.57894737 | 27 | 2.64E-05 | 1024279.211 | 118.154151 | 24.03 |
| 172_6.D | 14.1 | 1.7 | 12.0567376 | 27 | 2.41E-05 | 1120331.95 | 139.0894762 | 31.59 |
| 172_7.D | 6.36 | 0.55 | 8.64779874 | 23 | 3.09E-05 | 743614.6136 | 203.6447814 | 45.97 |
| 172_8.D | 5.5 | 0.54 | 9.81818182 | 16 | 3.25E-05 | 492307.6923 | 156.4777645 | 42.03 |
| 172_9.D | 13.7 | 0.87 | 6.35036496 | 12 | 2.30E-05 | 521739.1304 | 67.03983892 | 19.82 |
| 172_10.D | 5.6 | 0.42 | 7.5 | 13 | 2.37E-05 | 547829.7514 | 170.8248166 | 49.08 |
| 172_11.D | 3.92 | 0.31 | 7.90816327 | 17 | 2.43E-05 | 701030.9278 | 308.9324199 | 78.81 |
| 172_12.D | 5.56 | 0.51 | 9.17266187 | 19 | 3.59E-05 | 529247.9109 | 166.2767797 | 41.08 |
| 172_13.D | 11.58 | 0.8 | 6.90846287 | 21 | 3.18E-05 | 660792.9515 | 100.1930924 | 22.93 |
| 172_14.D | 15.9 | 1.3 | 8.17610063 | 22 | 2.82E-05 | 779312.7878 | 86.15286038 | 19.67 |
| 172_15.D | 18.4 | 1.2 | 6.52173913 | 27 | 3.50E-05 | 770988.0069 | 73.72323417 | 14.98 |
| 172_16.D | 7.79 | 0.78 | 10.012837 | 38 | 3.35E-05 | 1135005.974 | 252.8003102 | 48.19 |
| 172_18.D | 8.69 | 0.71 | 8.1703107 | 36 | 4.19E-05 | 859393.65 | 172.6646726 | 32.05 |
| 172_19.D | 12.97 | 0.71 | 5.47417116 | 31 | 4.32E-05 | 718091.2671 | 97.23453044 | 18.26 |
| 172_20.D | 7.89 | 0.89 | 11.2801014 | 24 | 3.30E-05 | 726392.2518 | 160.8880291 | 37.52 |
| 172_21.D | 7.7 | 0.84 | 10.9090909 | 21 | 4.06E-05 | 516859.4635 | 117.6986149 | 28.71 |
| 172_22.D | 7.25 | 0.54 | 7.44827586 | 21 | 3.91E-05 | 537496.8006 | 129.872172 | 29.95 |
| 172_23.D | 7.46 | 0.6 | 8.04289544 | 18 | 1.93E-05 | 934579.4393 | 217.9591339 | 54.28 |
| 172_24.D | 4.1 | 0.31 | 7.56097561 | 10 | 1.95E-05 | 514138.8175 | 218.166069 | 70.93 |
| 172_25.D | 7.81 | 0.55 | 7.04225352 | 13 | 2.49E-05 | 522718.1343 | 117.3593247 | 33.58 |
| 172_26.D | 19.4 | 1.8 | 9.27835052 | 12 | 1.96E-05 | 611932.6874 | 55.57619456 | 16.85 |
| 172_28.D | 34.3 | 2.4 | 6.99708455 | 35 | 1.75E-05 | 2004581.901 | 102.5957752 | 18.77 |
| 172_29.D | 8.55 | 0.58 | 6.78362573 | 17 | 2.28E-05 | 746268.6567 | 152.62931 | 38.44 |
| 172_30.D | 13.76 | 0.94 | 6.83139535 | 18 | 2.45E-05 | 734993.8751 | 93.83415828 | 23.03 |
| 172_31.D | 14.4 | 1.4 | 9.72222222 | 14 | 1.61E-05 | 870646.7662 | 106.1110374 | 30.18 |
| 172_32.D | 8.92 | 0.51 | 5.71748879 | 6 | 9.67E-06 | 620411.5397 | 121.9163921 | 50.26 |
| 172_34.D | 7.59 | 0.68 | 8.95915679 | 14 | 2.07E-05 | 677966.1017 | 156.1549738 | 44.02 |
| 172_35.D | 11.8 | 1.1 | 9.3220339 | 10 | 1.44E-05 | 696378.8301 | 103.5928235 | 34.15 |
| 172_36.D | 12.1 | 1.4 | 11.5702479 | 10 | 1.67E-05 | 598086.1244 | 86.87781796 | 29.25 |
| 172_38.D | 4.73 | 0.31 | 6.55391121 | 18 | 2.81E-05 | 641482.5374 | 235.6257701 | 57.64 |
| 172_39.D | 11.9 | 1.1 | 9.24369748 | 20 | 2.81E-05 | 711997.152 | 105.01453 | 25.41 |
| 172_40.D | 19.3 | 1.5 | 7.77202073 | 15 | 1.34E-05 | 1116071.429 | 101.5244526 | 27.38 |
| 172_41.D | 9.26 | 0.7 | 7.55939525 | 12 | 1.72E-05 | 697674.4186 | 131.9621552 | 39.38 |
| 172_42.D | 8.96 | 0.83 | 9.26339286 | 11 | 1.39E-05 | 793078.5869 | 154.7551727 | 48.81 |
| 172_43.D | 6.34 | 0.55 | 8.67507886 | 8 | 1.92E-05 | 416883.7936 | 115.3176553 | 41.98 |
| 172_44.D | 13.1 | 1.1 | 8.39694656 | 13 | 1.66E-05 | 782661.0476 | 104.8638052 | 30.39 |
| 172_45.D | 14.6 | 1.1 | 7.53424658 | 19 | 1.84E-05 | 1032047.8 | 123.8874856 | 29.92 |
| Name | 238U Dur (ppm) | SD (ppm) | RF | Ns | Area (cm^2) | rho s | t (Ma) | SD t (Ma) |
| 198_1.D | 5.03 | 0.33 | 6.56063618 | 7 | 2.15E-05 | 325430.0325 | 132.0647361 | 50.66 |
| 198_2.D | 4.99 | 0.45 | 9.01803607 | 14 | 2.43E-05 | 576843.8401 | 234.100106 | 66.03 |
| 198_3.D | 33.4 | 2.7 | 8.08383234 | 17 | 1.61E-05 | 1055245.189 | 64.82980397 | 16.57 |
| 198_4.D | 6.85 | 0.46 | 6.71532847 | 22 | 4.13E-05 | 532687.6513 | 158.4121314 | 35.41 |
| 198_9.D | 36.7 | 2.3 | 6.26702997 | 19 | 1.68E-05 | 1128936.423 | 63.12895536 | 15.01 |
| 198_11.D | 20.8 | 1.3 | 6.25 | 27 | 3.14E-05 | 859872.6115 | 84.69700548 | 17.14 |
| 198_15.D | 3.43 | 0.36 | 10.4956268 | 12 | 2.39E-05 | 502302.2185 | 295.1510313 | 90.66 |
| 198_16.D | 2.87 | 0.24 | 8.36236934 | 1 | 1.29E-05 | 77459.33385 | 55.4213463 | 55.61 |
| 198_19.D | 5.83 | 0.36 | 6.17495712 | 7 | 2.82E-05 | 248491.3028 | 87.30754601 | 33.44 |
| Name | 238U Dur (ppm) | SD (ppm) | RF | Ns | Area (cm^2) | rho s | t (Ma) | SD t (Ma) |
| 223_1.D | 70.3 | 8.9 | 12.6600284 | 98 | 3.94E-05 | 2490470.14 | 72.64905159 | 11.77 |
| 223_2.D | 23.9 | 1.8 | 7.53138075 | 44 | 1.74E-05 | 2522935.78 | 214.1063668 | 36.08 |
| 223_3.D | 39.7 | 7.9 | 19.8992443 | 61 | 3.18E-05 | 1916431.04 | 98.79251269 | 23.38 |
| 223_4.D | 19.9 | 1.2 | 6.03015075 | 58 | 3.76E-05 | 1544607.19 | 158.1179969 | 22.85 |
| 223_5.D | 18.2 | 1.4 | 7.69230769 | 45 | 2.93E-05 | 1537935.748 | 171.9550922 | 28.85 |
| 223_6.D | 19.5 | 1.2 | 6.15384615 | 58 | 3.66E-05 | 1586867.305 | 165.6786634 | 24.03 |
| 223_7.D | 18.1 | 1.1 | 6.07734807 | 49 | 2.63E-05 | 1860288.535 | 208.5504801 | 32.38 |
| 223_8.D | 25 | 2.2 | 8.8 | 41 | 3.40E-05 | 1204465.335 | 98.6011772 | 17.68 |
| 223_9.D | 131 | 12 | 9.16030534 | 178 | 3.73E-05 | 4774678.112 | 74.73197181 | 8.85 |
| 223_10.D | 13.5 | 1.1 | 8.14814815 | 41 | 2.79E-05 | 1470060.954 | 220.748551 | 38.89 |
| 223_11.D | 37.2 | 2.8 | 7.52688172 | 108 | 6.54E-05 | 1652639.633 | 90.9746838 | 11.11 |
| 223_12.D | 45.8 | 4.3 | 9.38864629 | 136 | 3.37E-05 | 4040404.04 | 179.4135245 | 22.81 |
| 223_13.D | 16.7 | 2 | 11.9760479 | 34 | 2.37E-05 | 1435204.728 | 174.842864 | 36.57 |
| 223_14.D | 33.8 | 2.7 | 7.98816568 | 82 | 3.88E-05 | 2113946.894 | 127.7090894 | 17.41 |
| 223_15.D | 21 | 1.6 | 7.61904762 | 79 | 3.43E-05 | 2301194.291 | 222.1180666 | 30.18 |
| 223_16.D | 19.6 | 1.2 | 6.12244898 | 54 | 3.48E-05 | 1552616.446 | 161.3302429 | 24.07 |
| 223_17.D | 19.2 | 1.4 | 7.29166667 | 37 | 2.86E-05 | 1295971.979 | 137.7210763 | 24.77 |
| 223_18.D | 21.8 | 1.5 | 6.88073394 | 65 | 3.42E-05 | 1901696.899 | 177.4383556 | 25.17 |
| 223_19.D | 25.6 | 2.4 | 9.375 | 45 | 3.62E-05 | 1243437.414 | 99.39963823 | 17.5 |
| 223_20.D | 52.9 | 5.1 | 9.64083176 | 68 | 3.15E-05 | 2160788.052 | 83.69271863 | 12.97 |
| 223_21.D | 34.7 | 3.3 | 9.51008646 | 69 | 4.33E-05 | 1592797.784 | 93.97561385 | 14.42 |
| 223_22.D | 11.16 | 0.9 | 8.06451613 | 23 | 2.77E-05 | 831525.6688 | 151.8586326 | 33.95 |
| 223_23.D | 14.5 | 0.74 | 5.10344828 | 43 | 4.88E-05 | 881689.5633 | 124.1969201 | 19.97 |
| 223_24.D | 26.4 | 1.5 | 5.68181818 | 79 | 4.05E-05 | 1951099.037 | 150.6414777 | 18.99 |
| 223_25.D | 68.9 | 5.4 | 7.83744557 | 88 | 3.39E-05 | 2595870.206 | 77.23478069 | 10.22 |
| 223_26.D | 13.9 | 1.3 | 9.35251799 | 63 | 4.70E-05 | 1340710.79 | 195.910524 | 30.74 |
| 223_27.D | 32.6 | 3.5 | 10.7361963 | 27 | 1.90E-05 | 1424802.111 | 89.51003617 | 19.73 |
| 223_28.D | 16.9 | 1.4 | 8.28402367 | 62 | 2.48E-05 | 2496979.46 | 297.7242775 | 45.14 |
| 223_29.D | 56.2 | 3.8 | 6.76156584 | 86 | 3.55E-05 | 2425267.908 | 88.38855847 | 11.25 |
| 223_30.D | 99 | 10 | 10.1010101 | 83 | 3.56E-05 | 2332771.22 | 48.41261819 | 7.22 |
| 223_31.D | 158 | 13 | 8.2278481 | 91 | 2.50E-05 | 3645833.333 | 47.41274098 | 6.32 |
| 223_32.D | 23.5 | 1.4 | 5.95744681 | 35 | 2.27E-05 | 1541850.22 | 133.9088303 | 24 |
| 223_34.D | 33.6 | 3.1 | 9.22619048 | 44 | 1.88E-05 | 2335456.476 | 141.7755877 | 25.06 |
| 223_35.D | 45.5 | 4.7 | 10.3296703 | 42 | 1.89E-05 | 2219873.15 | 99.83957934 | 18.54 |
| 223_38.D | 103.3 | 8.3 | 8.03484995 | 145 | 2.80E-05 | 5174875.089 | 102.4934006 | 11.84 |
| 223_40.D | 10.21 | 0.78 | 7.63956905 | 15 | 2.31E-05 | 650195.0585 | 130.0123222 | 35.01 |
| 223_41.D | 23.4 | 1.8 | 7.69230769 | 57 | 3.22E-05 | 1769636.759 | 154.1062605 | 23.6 |
| 223_42.D | 36.4 | 4.1 | 11.2637363 | 53 | 1.76E-05 | 3018223.235 | 168.7741132 | 29.98 |
| Name | 238U Dur (ppm) | SD (ppm) | RF | Ns | Area (cm^2) | rho s | t (Ma) | SD t (Ma) |
| 252_1.D | 8.09 | 0.68 | 8.40543881 | 22 | 1.37E-05 | 1601164.483 | 377.8961266 | 86.6 |
| 252_2.D | 8.81 | 0.51 | 5.78887628 | 33 | 3.23E-05 | 1021988.232 | 224.1680056 | 41.12 |
| 252_4.D | 1.73 | 0.14 | 8.09248555 | 7 | 1.46E-05 | 479452.0548 | 523.159264 | 202.22 |
| 252_5.D | 2.54 | 0.26 | 10.2362205 | 10 | 1.53E-05 | 653167.8641 | 486.8173945 | 161.81 |
| 252_6.D | 5.45 | 0.48 | 8.80733945 | 27 | 2.43E-05 | 1110654.052 | 388.7740452 | 82.28 |
| 252_10.D | 9.13 | 0.76 | 8.32420591 | 27 | 1.65E-05 | 1634382.567 | 342.738793 | 71.87 |
| 252_11.D | 5.14 | 0.27 | 5.25291829 | 13 | 1.96E-05 | 664282.0644 | 249.2544055 | 70.36 |
| 252_12.D | 7.95 | 0.57 | 7.16981132 | 16 | 1.85E-05 | 865332.6122 | 210.5622293 | 54.76 |
| 252_13.D | 5.68 | 0.5 | 8.8028169 | 12 | 2.13E-05 | 563115.908 | 192.0615485 | 57.96 |
| 252_14.D | 3.12 | 0.22 | 7.05128205 | 9 | 1.45E-05 | 620261.8884 | 379.533251 | 129.31 |
| 252_15.D | 1.68 | 0.18 | 10.7142857 | 6 | 1.59E-05 | 377121.3074 | 426.9597183 | 180.21 |
| 252_16.D | 7.37 | 0.57 | 7.73405699 | 6 | 9.69E-06 | 618939.5502 | 163.0621042 | 67.75 |
| 252_18.D | 3.63 | 0.35 | 9.64187328 | 17 | 2.12E-05 | 803782.5059 | 421.3453754 | 109.97 |
| 252_19.D | 4.89 | 0.66 | 13.4969325 | 14 | 1.94E-05 | 720535.2548 | 283.4267207 | 84.86 |
| 252_20.D | 1.94 | 0.2 | 10.3092784 | 9 | 2.64E-05 | 340651.022 | 336.3605263 | 117.36 |
| 252_24.D | 6.77 | 0.84 | 12.4076809 | 11 | 1.23E-05 | 894308.9431 | 254.6647907 | 83.03 |
| 252_25.D | 7.1 | 2.5 | 35.2112676 | 8 | 1.96E-05 | 408788.9627 | 112.2357684 | 56 |
| 252_26.D | 2.92 | 0.26 | 8.90410959 | 6 | 1.61E-05 | 373366.5215 | 246.657316 | 103.06 |
| 252_27.D | 6.82 | 0.61 | 8.94428152 | 8 | 1.27E-05 | 632411.0672 | 179.8128125 | 65.58 |
| 252_28.D | 6.03 | 0.78 | 12.9353234 | 26 | 2.47E-05 | 1052631.579 | 334.4423855 | 78.57 |
| 252_29.D | 8.3 | 1.1 | 13.253012 | 10 | 1.17E-05 | 852514.919 | 198.8768027 | 68.19 |
| 252_30.D | 4.96 | 0.23 | 4.63709677 | 12 | 1.84E-05 | 651112.3169 | 253.1029402 | 74 |
| 252_31.D | 4.54 | 0.42 | 9.25110132 | 23 | 2.34E-05 | 981228.6689 | 411.5789973 | 93.89 |
| 252_32.D | 19.3 | 1.9 | 9.84455959 | 3 | 1.25E-05 | 240384.6154 | 24.4456879 | 14.32 |
| 252_33.D | 4.03 | 0.29 | 7.19602978 | 15 | 2.25E-05 | 666370.502 | 317.2181535 | 85.03 |
| 252_35.D | 3.37 | 0.23 | 6.82492582 | 8 | 1.97E-05 | 406091.3706 | 232.7063262 | 83.79 |
| 252_36.D | 2.48 | 0.16 | 6.4516129 | 2 | 8.82E-06 | 226834.5242 | 177.3963966 | 125.96 |
| 252_38.D | 2.84 | 0.22 | 7.74647887 | 7 | 1.58E-05 | 443599.493 | 300.0562835 | 115.77 |
| 252_39.D | 2.27 | 0.27 | 11.8942731 | 5 | 9.00E-06 | 555864.3691 | 464.3898657 | 214.9 |
| Name | 238U Dur (ppm) | SD (ppm) | RF | Ns | Area (cm^2) | rho s | t (Ma) | SD t (Ma) |
| 270_1.D | 3.53 | 0.32 | 9.06515581 | 7 | 2.44E-05 | 286650.2867 | 165.3293932 | 64.26 |
| 270_2.D | 7.81 | 0.58 | 7.42637644 | 11 | 1.86E-05 | 592034.4456 | 154.4670135 | 47.97 |
| 270_3.D | 2.04 | 0.26 | 12.745098 | 4 | 1.82E-05 | 220022.0022 | 218.6766544 | 112.83 |
| 270_4.D | 3.95 | 0.72 | 18.2278481 | 6 | 2.68E-05 | 224047.7969 | 115.9274101 | 51.83 |
| 270_5.D | 1.68 | 0.27 | 16.0714286 | 6 | 3.30E-05 | 182094.0819 | 219.7439217 | 96.41 |
| 270_6.D | 2.01 | 0.26 | 12.9353234 | 5 | 2.40E-05 | 208507.0892 | 210.4599957 | 97.98 |
| 270_7.D | 4.88 | 0.25 | 5.12295082 | 7 | 4.22E-05 | 165876.7773 | 69.7217836 | 26.59 |
| 270_8.D | 1.87 | 0.15 | 8.02139037 | 3 | 2.89E-05 | 103950.104 | 113.632719 | 66.24 |
| 270_12.D | 7.58 | 0.61 | 8.0474934 | 8 | 2.30E-05 | 347826.087 | 93.94592589 | 34.06 |
| 270_13.D | 4.08 | 0.45 | 11.0294118 | 7 | 2.21E-05 | 316455.6962 | 158.0057454 | 62.21 |
| 270_14.D | 4.69 | 0.88 | 18.7633262 | 5 | 3.97E-05 | 126071.6087 | 55.19975336 | 26.77 |
| 270_15.D | 1.76 | 0.19 | 10.7954545 | 11 | 2.93E-05 | 375170.5321 | 425.270723 | 136.2 |
| 270_17.D | 10.5 | 1.1 | 10.4761905 | 12 | 3.08E-05 | 390243.9024 | 76.19580467 | 23.4 |
| 270_18.D | 2.6 | 0.24 | 9.23076923 | 5 | 2.67E-05 | 187265.9176 | 146.8529522 | 67.06 |
| 270_20.D | 11.88 | 0.7 | 5.89225589 | 18 | 2.23E-05 | 805729.6329 | 138.3747418 | 33.62 |
| 270_22.D | 3.96 | 0.41 | 10.3535354 | 15 | 3.83E-05 | 391644.9086 | 200.8025067 | 55.86 |
| 270_23.D | 2.12 | 0.32 | 15.0943396 | 2 | 1.33E-05 | 150150.1502 | 144.433851 | 104.43 |
| 270_24.D | 2.72 | 0.23 | 8.45588235 | 3 | 2.28E-05 | 131463.6284 | 98.91313772 | 57.72 |
| 270_25.D | 2.11 | 0.18 | 8.53080569 | 6 | 4.86E-05 | 123380.6292 | 119.477773 | 49.83 |
| 270_27.D | 3.36 | 0.19 | 5.6547619 | 4 | 1.26E-05 | 318471.3376 | 192.5669406 | 96.9 |
| 270_28.D | 2.51 | 0.2 | 7.96812749 | 5 | 1.60E-05 | 312304.8095 | 251.6255839 | 114.3 |
| 270_31.D | 5.96 | 0.5 | 8.38926174 | 7 | 2.94E-05 | 238500.8518 | 82.00340367 | 31.75 |
| 270_32.D | 6.19 | 0.39 | 6.30048465 | 10 | 2.09E-05 | 478468.8995 | 157.4712473 | 50.78 |
| 270_33.D | 3 | 0.18 | 6 | 13 | 3.37E-05 | 385871.1784 | 259.9493239 | 73.76 |
| 270_35.D | 7.32 | 0.4 | 5.46448087 | 4 | 1.79E-05 | 223463.6872 | 62.65231791 | 31.51 |
| 270_36.D | 4.19 | 0.59 | 14.0811456 | 7 | 3.00E-05 | 233022.6365 | 113.6848011 | 45.85 |
| 270_37.D | 7.31 | 0.72 | 9.8495212 | 8 | 2.08E-05 | 385542.1687 | 107.8622612 | 39.59 |
| 270_39.D | 4.06 | 0.38 | 9.35960591 | 16 | 2.50E-05 | 641282.5651 | 317.7813363 | 84.83 |
| Name | 238U Dur (ppm) | SD (ppm) | RF | Ns | Area (cm^2) | rho s | t (Ma) | SD t (Ma) |
| 278_1.D | 19.4 | 1.3 | 6.70103093 | 78 | 4.58E-05 | 1703800.786 | 153.5657166 | 20.2 |
| 278_2.D | 37.2 | 2.5 | 6.72043011 | 70 | 2.75E-05 | 2550091.075 | 120.1762479 | 16.48 |
| 278_3.D | 25.7 | 2.1 | 8.17120623 | 20 | 1.92E-05 | 1041124.414 | 71.28960649 | 16.97 |
| 278_4.D | 39.4 | 2.1 | 5.32994924 | 141 | 4.72E-05 | 2984758.679 | 132.6772372 | 13.22 |
| 278_5.D | 26.8 | 2.5 | 9.32835821 | 48 | 2.74E-05 | 1751185.699 | 114.6018531 | 19.7 |
| 278_6.D | 11.9 | 2.4 | 20.1680672 | 37 | 3.32E-05 | 1114122.252 | 163.5778264 | 42.56 |
| 278_7.D | 49 | 4.9 | 10 | 50 | 2.88E-05 | 1733703.19 | 62.30723395 | 10.79 |
| 278_8.D | 35.2 | 3.4 | 9.65909091 | 74 | 3.29E-05 | 2251292.972 | 112.1927961 | 16.96 |
| 278_9.D | 14 | 1.2 | 8.57142857 | 21 | 2.14E-05 | 980850.0701 | 122.7981671 | 28.79 |
| 278_10.D | 22.6 | 1.8 | 7.96460177 | 32 | 2.30E-05 | 1392515.231 | 108.1196741 | 20.96 |
| 278_12.D | 13.9 | 1.1 | 7.91366906 | 25 | 2.71E-05 | 922849.7601 | 116.425689 | 25.04 |
| 278_13.D | 39.3 | 2.8 | 7.12468193 | 74 | 2.48E-05 | 2982668.279 | 132.9191774 | 18.12 |
| 278_14.D | 8.81 | 0.6 | 6.81044268 | 23 | 2.93E-05 | 784180.0205 | 155.6136858 | 34.13 |
| 278_15.D | 39.4 | 2.7 | 6.85279188 | 70 | 3.68E-05 | 1904243.743 | 84.9613808 | 11.71 |
| 278_16.D | 35.2 | 3 | 8.52272727 | 60 | 2.46E-05 | 2437043.054 | 121.3630066 | 18.77 |
| 278_17.D | 34.5 | 3 | 8.69565217 | 89 | 2.88E-05 | 3092425.295 | 156.6936943 | 21.48 |
| 278_18.D | 37.2 | 2.7 | 7.25806452 | 92 | 2.80E-05 | 3289238.47 | 154.5947728 | 19.64 |
| 278_19.D | 34.6 | 3.2 | 9.24855491 | 31 | 2.27E-05 | 1368049.426 | 69.58887139 | 14.06 |
| 278_20.D | 14.4 | 1.7 | 11.8055556 | 21 | 2.30E-05 | 913440.6264 | 111.2818215 | 27.61 |
| 278_21.D | 32.5 | 3.1 | 9.53846154 | 67 | 2.67E-05 | 2507485.03 | 135.1003581 | 20.94 |
| 278_22.D | 9.36 | 0.83 | 8.86752137 | 23 | 1.53E-05 | 1501305.483 | 277.7561808 | 62.94 |
| 278_23.D | 21 | 2.7 | 12.8571429 | 18 | 1.40E-05 | 1282965.075 | 107.2110244 | 28.78 |
| 278_24.D | 17.7 | 1.4 | 7.90960452 | 23 | 1.98E-05 | 1159858.8 | 114.9251726 | 25.63 |
| 278_25.D | 37.9 | 2.8 | 7.3878628 | 36 | 1.65E-05 | 2180496.669 | 101.0111991 | 18.42 |
| 278_26.D | 16.5 | 1.5 | 9.09090909 | 26 | 2.56E-05 | 1014040.562 | 107.8434879 | 23.31 |
| 278_27.D | 16.8 | 1.8 | 10.7142857 | 14 | 1.92E-05 | 728407.9084 | 76.27004361 | 21.96 |
| 278_28.D | 16.5 | 1.4 | 8.48484848 | 28 | 1.57E-05 | 1780038.144 | 188.1278892 | 38.97 |
| 278_29.D | 13.6 | 1.5 | 11.0294118 | 10 | 1.38E-05 | 726744.186 | 93.87221149 | 31.44 |
| 278_30.D | 20.1 | 1.7 | 8.45771144 | 22 | 1.48E-05 | 1486486.486 | 129.5549043 | 29.72 |
| 278_31.D | 22.2 | 1.8 | 8.10810811 | 92 | 3.82E-05 | 2407746.663 | 189.1176792 | 24.98 |
| Name | 238U Dur (ppm) | SD (ppm) | RF | Ns | Area (cm^2) | rho s | t (Ma) | SD t (Ma) |
| 396_1.D | 28.7 | 2.3 | 8.01393728 | 73 | 2.51E-05 | 2907208.284 | 176.8012801 | 25.08 |
| 396_3.D | 24.6 | 1.8 | 7.31707317 | 19 | 1.30E-05 | 1466049.383 | 104.6033225 | 25.19 |
| 396_4.D | 25.9 | 1.5 | 5.79150579 | 32 | 1.70E-05 | 1884570.082 | 127.4885886 | 23.72 |
| 396_5.D | 29.3 | 2.5 | 8.53242321 | 32 | 1.90E-05 | 1683324.566 | 100.8691469 | 19.8 |
| 396_6.D | 12.2 | 1.1 | 9.01639344 | 24 | 1.37E-05 | 1755669.349 | 249.7487193 | 55.73 |
| 396_7.D | 21 | 1.8 | 8.57142857 | 54 | 3.26E-05 | 1654918.786 | 137.9628446 | 22.19 |
| 396_8.D | 29.7 | 3 | 10.1010101 | 51 | 2.48E-05 | 2054794.521 | 121.2776083 | 20.94 |
| 396_9.D | 29.6 | 1.6 | 5.40540541 | 54 | 2.42E-05 | 2228642.179 | 131.8740067 | 19.31 |
| 396_10.D | 30.1 | 2.7 | 8.97009967 | 39 | 1.68E-05 | 2317290.553 | 134.8109872 | 24.74 |
| 396_11.D | 29.8 | 1.7 | 5.70469799 | 77 | 3.79E-05 | 2030590.717 | 119.4637373 | 15.22 |
| 396_12.D | 17.6 | 1.8 | 10.2272727 | 44 | 3.40E-05 | 1294879.341 | 128.8925952 | 23.48 |
| 396_13.D | 21.8 | 1.7 | 7.79816514 | 60 | 3.27E-05 | 1832620.648 | 147.0662615 | 22.18 |
| 396_14.D | 22.3 | 1.7 | 7.62331839 | 59 | 3.09E-05 | 1910621.762 | 149.8554402 | 22.61 |
| 396_15.D | 30.5 | 2.4 | 7.86885246 | 96 | 3.96E-05 | 2422407.267 | 139.0323664 | 17.92 |
| 396_16.D | 31.6 | 2.5 | 7.91139241 | 62 | 2.54E-05 | 2440944.882 | 135.2592674 | 20.24 |
| 396_17.D | 32.8 | 2.8 | 8.53658537 | 75 | 3.42E-05 | 2194907.814 | 117.3394856 | 16.85 |
| 396_18.D | 33.4 | 3.1 | 9.28143713 | 101 | 3.13E-05 | 3225806.452 | 168.677772 | 22.95 |
| 396_19.D | 30.7 | 2.5 | 8.14332248 | 58 | 2.71E-05 | 2144177.449 | 122.4200126 | 18.91 |
| 396_20.D | 26.7 | 1.8 | 6.74157303 | 59 | 2.65E-05 | 2229780.801 | 146.1100913 | 21.42 |
| 396_21.D | 22.9 | 1.4 | 6.11353712 | 16 | 1.30E-05 | 1229823.213 | 94.33782798 | 24.28 |
| 396_22.D | 20.7 | 2 | 9.66183575 | 57 | 2.24E-05 | 2542372.881 | 213.7514683 | 35.04 |
| 396_23.D | 25.2 | 1.9 | 7.53968254 | 30 | 1.85E-05 | 1620745.543 | 112.8155271 | 22.28 |
| 396_24.D | 27 | 1.9 | 7.03703704 | 51 | 2.07E-05 | 2469733.656 | 159.8639454 | 25.05 |
| 396_26.D | 30.9 | 2.1 | 6.7961165 | 49 | 2.09E-05 | 2344497.608 | 132.8825012 | 21.02 |
| 396_28.D | 26.1 | 1.4 | 5.36398467 | 52 | 2.30E-05 | 2261852.98 | 151.5545794 | 22.53 |
| 396_29.D | 29 | 2 | 6.89655172 | 81 | 3.09E-05 | 2620511.161 | 157.9490273 | 20.66 |
| 396_30.D | 31.8 | 2.2 | 6.91823899 | 66 | 2.33E-05 | 2837489.252 | 155.9919676 | 22.03 |
| 396_31.D | 25.9 | 1.7 | 6.56370656 | 49 | 2.25E-05 | 2177777.778 | 147.0989804 | 23.13 |
| 396_32.D | 27.5 | 1.8 | 6.54545455 | 25 | 1.38E-05 | 1806358.382 | 115.1980992 | 24.24 |
| 396_33.D | 28.8 | 3 | 10.4166667 | 48 | 2.24E-05 | 2146690.519 | 130.5664844 | 23.24 |
| 396_34.D | 27 | 2.7 | 10 | 57 | 2.43E-05 | 2348578.492 | 152.1134492 | 25.25 |
| 396_35.D | 24.1 | 1.9 | 7.88381743 | 40 | 2.12E-05 | 1883239.171 | 136.8144617 | 24.17 |
| 396_36.D | 28.1 | 2.6 | 9.25266904 | 34 | 2.30E-05 | 1481481.481 | 92.62461703 | 18.05 |
| 396_37.D | 26.3 | 1.8 | 6.84410646 | 46 | 1.60E-05 | 2882205.514 | 191.063463 | 31.06 |
| 396_38.D | 14.7 | 1.1 | 7.4829932 | 34 | 3.00E-05 | 1132955.681 | 134.9590079 | 25.25 |
| 396_39.D | 28.1 | 1.7 | 6.04982206 | 43 | 2.18E-05 | 1976102.941 | 123.2551557 | 20.22 |
| 396_40.D | 28 | 2.9 | 10.3571429 | 25 | 1.50E-05 | 1671122.995 | 104.7555949 | 23.59 |
| 396_41.D | 28.2 | 2.7 | 9.57446809 | 29 | 1.75E-05 | 1655251.142 | 103.0385234 | 21.53 |
| 396_42.D | 10.66 | 0.84 | 7.87992495 | 15 | 2.59E-05 | 579374.2758 | 95.46465837 | 25.77 |
| Name | 238U Dur (ppm) | SD (ppm) | RF | Ns | Area (cm^2) | rho s | t (Ma) | SD t (Ma) |
| 446_1.D | 17.6 | 1.3 | 7.38636364 | 103 | 3.12E-05 | 3297055.058 | 323.250831 | 39.81 |
| 446_2.D | 12.97 | 0.9 | 6.93909021 | 24 | 1.09E-05 | 2209944.751 | 294.6713728 | 63.53 |
| 446_3.D | 10.96 | 0.57 | 5.20072993 | 26 | 1.48E-05 | 1761517.615 | 278.3095894 | 56.47 |
| 446_4.D | 13.35 | 0.92 | 6.89138577 | 51 | 2.21E-05 | 2305605.787 | 298.5846413 | 46.6 |
| 446_6.D | 29.1 | 2 | 6.87285223 | 48 | 1.76E-05 | 2727272.727 | 163.7451578 | 26.18 |
| 446_7.D | 14.52 | 0.88 | 6.06060606 | 50 | 2.58E-05 | 1937233.63 | 231.8685255 | 35.68 |
| 446_8.D | 16.3 | 1.2 | 7.36196319 | 43 | 2.04E-05 | 2104747.92 | 224.5366976 | 38.02 |
| 446_9.D | 17.4 | 1.4 | 8.04597701 | 29 | 1.63E-05 | 1783517.835 | 178.8748138 | 36.2 |
| 446_10.D | 16.5 | 1.1 | 6.66666667 | 63 | 2.71E-05 | 2322152.599 | 244.3488178 | 34.83 |
| 446_11.D | 12.6 | 1.2 | 9.52380952 | 20 | 1.59E-05 | 1259445.844 | 174.4930812 | 42.41 |
| 446_12.D | 9.17 | 0.74 | 8.0697928 | 21 | 2.00E-05 | 1048427.359 | 199.2057193 | 46.35 |
| 446_13.D | 12.8 | 1.5 | 11.71875 | 27 | 1.36E-05 | 1986754.967 | 268.9694289 | 60.6 |
| 446_14.D | 10.23 | 0.85 | 8.30889541 | 17 | 9.22E-06 | 1844217.835 | 311.3625977 | 79.83 |
| 446_15.D | 15.3 | 1.3 | 8.49673203 | 28 | 1.42E-05 | 1977401.13 | 224.7353868 | 46.57 |
| 446_16.D | 19.3 | 2 | 10.3626943 | 29 | 2.21E-05 | 1315192.744 | 119.4708134 | 25.41 |
| 446_17.D | 13.9 | 1.3 | 9.35251799 | 27 | 1.12E-05 | 2417188.899 | 300.6014055 | 64.32 |
| 446_18.D | 19.3 | 1.2 | 6.21761658 | 53 | 2.80E-05 | 1892181.364 | 171.1931432 | 25.81 |
| 446_20.D | 17.5 | 1.7 | 9.71428571 | 28 | 1.98E-05 | 1412002.017 | 141.2186161 | 30.01 |
| 446_21.D | 9.92 | 0.66 | 6.65322581 | 28 | 1.84E-05 | 1524224.279 | 266.3151145 | 53.36 |
| 446_22.D | 10.65 | 0.79 | 7.41784038 | 30 | 1.79E-05 | 1675041.876 | 272.4744296 | 53.7 |
| 446_24.D | 14.9 | 1.8 | 12.0805369 | 16 | 1.18E-05 | 1360544.218 | 159.5878784 | 44.31 |
| 446_25.D | 12.6 | 1.5 | 11.9047619 | 18 | 1.26E-05 | 1425178.147 | 197.1071843 | 52.05 |
| 446_26.D | 9.4 | 0.8 | 8.5106383 | 5 | 6.59E-06 | 759070.8972 | 141.3335554 | 64.34 |
| 446_27.D | 15.3 | 1.4 | 9.1503268 | 73 | 2.85E-05 | 2565003.514 | 290.0349912 | 43.09 |
| 446_28.D | 14.4 | 1.2 | 8.33333333 | 44 | 2.19E-05 | 2007299.27 | 242.0641683 | 41.7 |
| 446_29.D | 16 | 1.3 | 8.125 | 96 | 3.09E-05 | 3111831.442 | 335.2844392 | 43.74 |
| Name | 238U Dur (ppm) | SD (ppm) | RF | Ns | Area (cm^2) | rho s | t (Ma) | SD t (Ma) |
| 598_1.D | 20 | 1.4 | 7 | 52 | 4.87E-05 | 1067104.453 | 93.72946717 | 14.56 |
| 598_2.D | 22.4 | 1.8 | 8.03571429 | 37 | 1.88E-05 | 1973333.333 | 154.033015 | 28.19 |
| 598_3.D | 23.3 | 1.9 | 8.15450644 | 42 | 2.92E-05 | 1439835.447 | 108.4325286 | 18.92 |
| 598_4.D | 12.99 | 0.88 | 6.77444188 | 26 | 2.43E-05 | 1069958.848 | 144.1299304 | 29.91 |
| 598_6.D | 12.38 | 0.86 | 6.94668821 | 48 | 2.67E-05 | 1797752.809 | 251.9731634 | 40.36 |
| 598_7.D | 8.36 | 0.85 | 10.1674641 | 30 | 2.18E-05 | 1376778.339 | 285.024147 | 59.56 |
| 598_8.D | 16.4 | 1.7 | 10.3658537 | 20 | 2.09E-05 | 958772.7709 | 102.6290976 | 25.29 |
| 598_9.D | 14.5 | 2.1 | 14.4827586 | 45 | 2.57E-05 | 1748931.209 | 209.9774961 | 43.64 |
| 598_10.D | 8.29 | 0.76 | 9.16767189 | 37 | 3.32E-05 | 1115129.596 | 233.7404065 | 44 |
| 598_11.D | 21 | 1.9 | 9.04761905 | 48 | 2.32E-05 | 2068965.517 | 172.0231082 | 29.3 |
| 598_12.D | 16 | 1.4 | 8.75 | 31 | 2.99E-05 | 1037483.266 | 113.7325352 | 22.72 |
| 598_13.D | 7.6 | 0.66 | 8.68421053 | 35 | 3.54E-05 | 987863.3926 | 226.0000657 | 42.95 |
| 598_14.D | 22.3 | 2.1 | 9.41704036 | 58 | 2.92E-05 | 1989708.405 | 155.9839712 | 25.2 |
| 598_15.D | 21.9 | 1.5 | 6.84931507 | 20 | 2.47E-05 | 809061.4887 | 65.04364789 | 15.21 |
| 598_16.D | 19 | 1.5 | 7.89473684 | 43 | 3.08E-05 | 1397011.046 | 128.8131669 | 22.12 |
| 598_17.D | 7.21 | 0.49 | 6.7961165 | 25 | 3.30E-05 | 757346.2587 | 183.2450724 | 38.71 |
| 598_18.D | 30 | 2.6 | 8.66666667 | 27 | 2.35E-05 | 1146983.857 | 67.30190036 | 14.21 |
| 598_19.D | 17.7 | 1.3 | 7.34463277 | 33 | 3.27E-05 | 1010719.755 | 100.2619395 | 18.94 |
| 598_20.D | 13.5 | 1.4 | 10.3703704 | 22 | 3.42E-05 | 643839.6254 | 83.84494213 | 19.88 |
| 598_21.D | 14.2 | 1 | 7.04225352 | 33 | 4.18E-05 | 790040.6991 | 97.70707554 | 18.35 |
| 598_22.D | 13.8 | 2.2 | 15.942029 | 13 | 3.75E-05 | 346759.1358 | 44.31141849 | 14.18 |
| 598_24.D | 6.91 | 0.58 | 8.39363242 | 25 | 2.62E-05 | 955657.4924 | 240.1974361 | 52.1 |
| 598_25.D | 6.5 | 1 | 15.3846154 | 36 | 3.07E-05 | 1171112.557 | 311.1867593 | 70.58 |
| 598_26.D | 21.4 | 2.6 | 12.1495327 | 43 | 2.96E-05 | 1450742.24 | 118.8575923 | 23.17 |
| 598_27.D | 6.56 | 0.52 | 7.92682927 | 28 | 4.60E-05 | 608828.0061 | 162.1723725 | 33.23 |
| 598_28.D | 17.4 | 2.5 | 14.3678161 | 20 | 2.91E-05 | 686813.1868 | 69.47162171 | 18.46 |
| 598_29.D | 18.7 | 1.6 | 8.55614973 | 29 | 2.36E-05 | 1227253.491 | 115.0986206 | 23.53 |
| 598_30.D | 10.8 | 1.5 | 13.8888889 | 57 | 2.40E-05 | 2371048.253 | 377.2315026 | 72.4 |
| 598_31.D | 5.33 | 0.35 | 6.56660413 | 33 | 5.90E-05 | 559416.8503 | 183.0991627 | 34.07 |
| 598_32.D | 7.37 | 0.61 | 8.27679783 | 11 | 2.21E-05 | 496838.3017 | 118.2007466 | 36.96 |
| 598_33.D | 10.4 | 1.1 | 10.5769231 | 13 | 2.00E-05 | 650976.4647 | 109.8215759 | 32.6 |
| 598_34.D | 11.8 | 1.1 | 9.3220339 | 29 | 3.27E-05 | 887121.4439 | 131.6795127 | 27.36 |
| 598_35.D | 19.3 | 1.7 | 8.80829016 | 32 | 3.58E-05 | 894104.4985 | 81.46000174 | 16.09 |
| 598_36.D | 17.4 | 1.7 | 9.77011494 | 23 | 1.84E-05 | 1247288.503 | 125.6145049 | 28.93 |
| 598_37.D | 9.25 | 0.95 | 10.2702703 | 19 | 3.23E-05 | 588235.2941 | 111.5594492 | 28.04 |
| 598_38.D | 17.5 | 1.3 | 7.42857143 | 27 | 2.50E-05 | 1082164.329 | 108.5063902 | 22.38 |
| 598_39.D | 24.7 | 2.6 | 10.5263158 | 54 | 2.67E-05 | 2023229.674 | 143.3412383 | 24.66 |
| 598_40.D | 27.5 | 2.8 | 10.1818182 | 51 | 3.76E-05 | 1357104.843 | 86.73930356 | 15.02 |
| 598_41.D | 17.7 | 2.1 | 11.8644068 | 34 | 3.40E-05 | 999118.4249 | 99.11990651 | 20.67 |
| 598_42.D | 30.1 | 2.2 | 7.3089701 | 28 | 2.97E-05 | 942126.5141 | 55.14980712 | 11.17 |

Apatite Fission Track Data Table Description

238 U Dur – The uranium concentration of each measured grain in ppm

SD - Standard deviation of the uranium concentration of each measured grain in ppm

RF – The relative uncertainty of individual grains

Ns – Number of spontaneous tracks in each analysed grain

Area – The area from which the spontaneous tracks were counted

rho’s – The density of spontaneous fission tracks within the counted area

t (Ma) - Calculated age for each individual grain in millions of years

SD (t) – Standard deviation of the age calculated from each individual grain in millions of years

Supplementary Fig S1


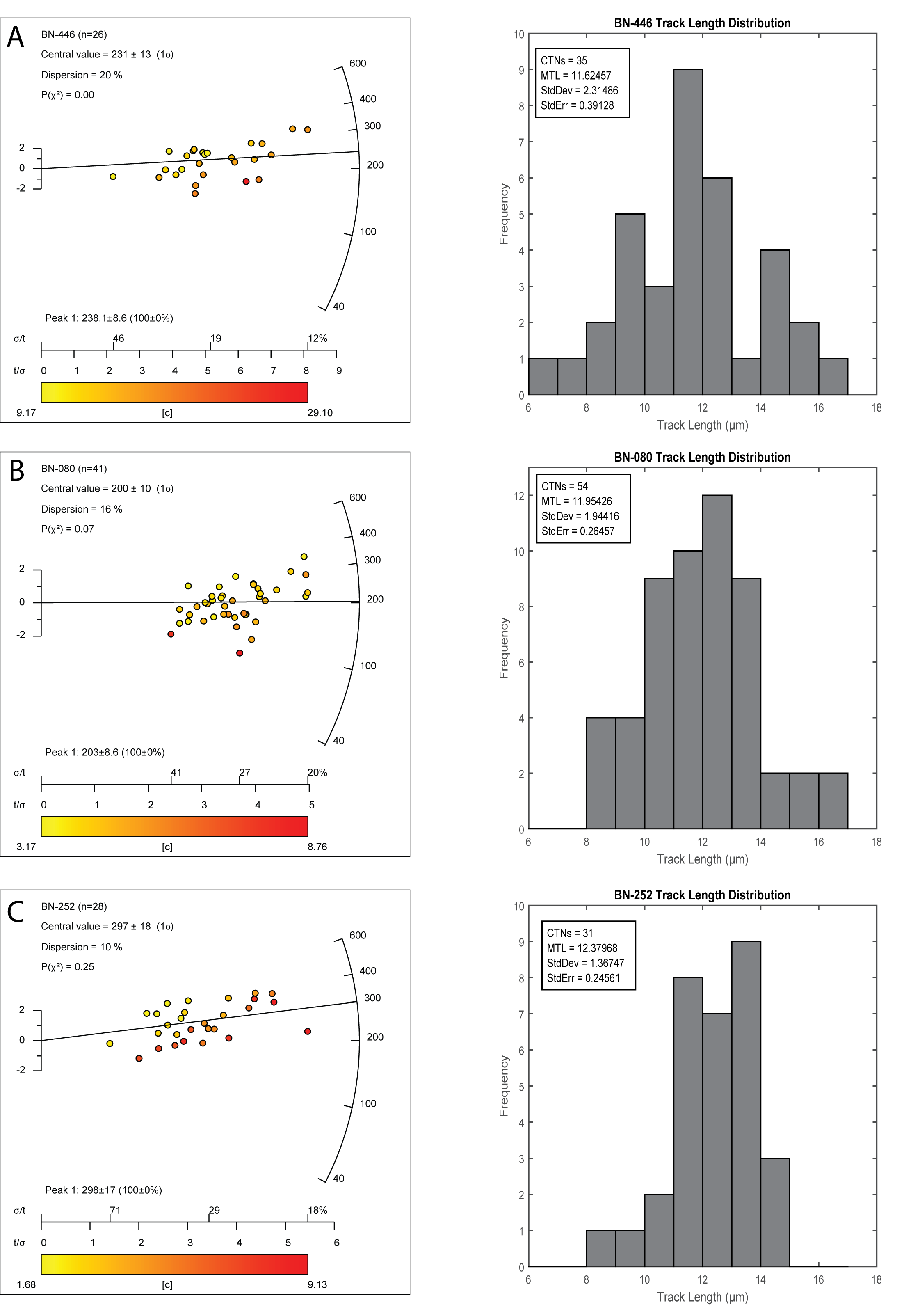


Supplementary Fig S2


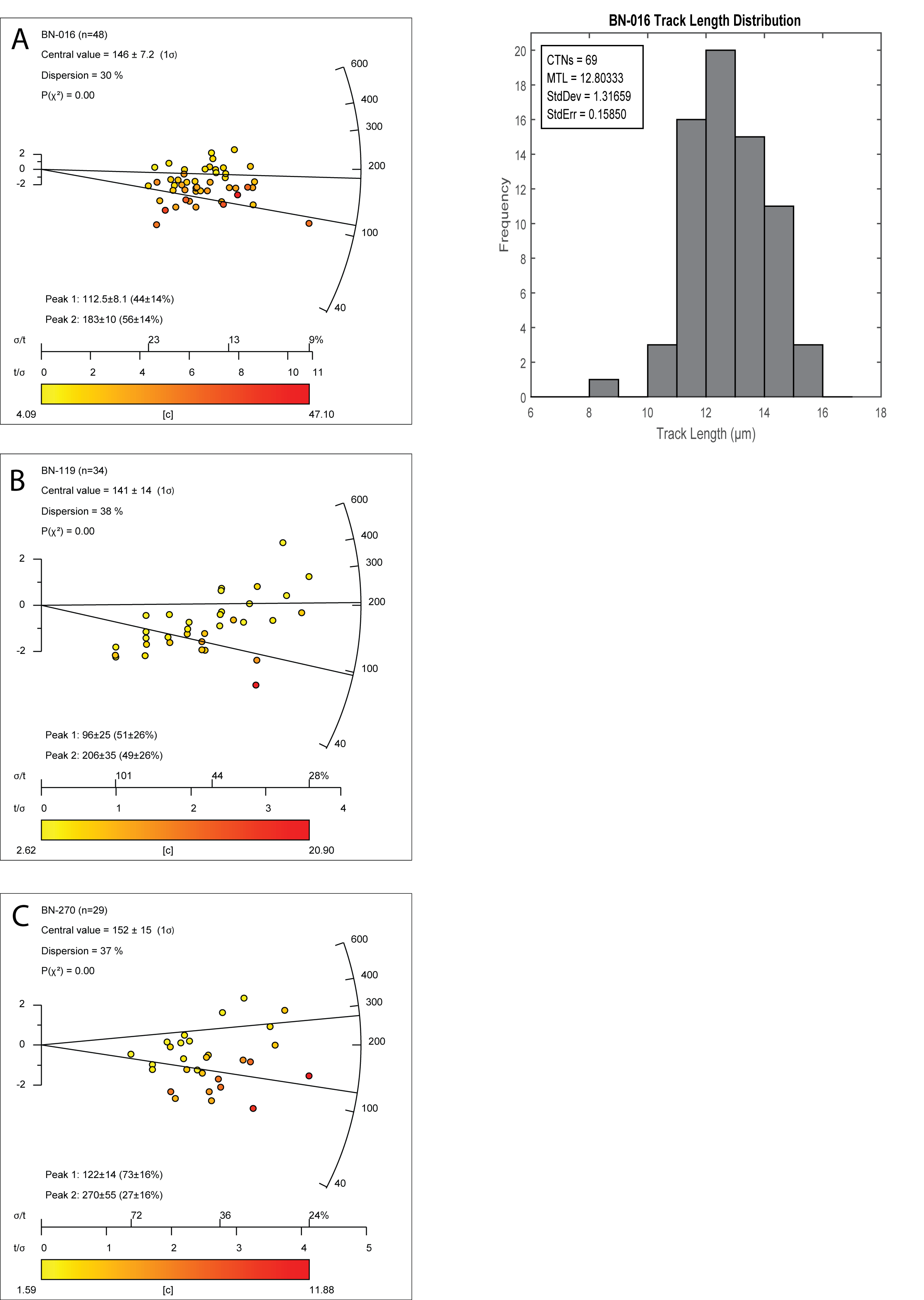

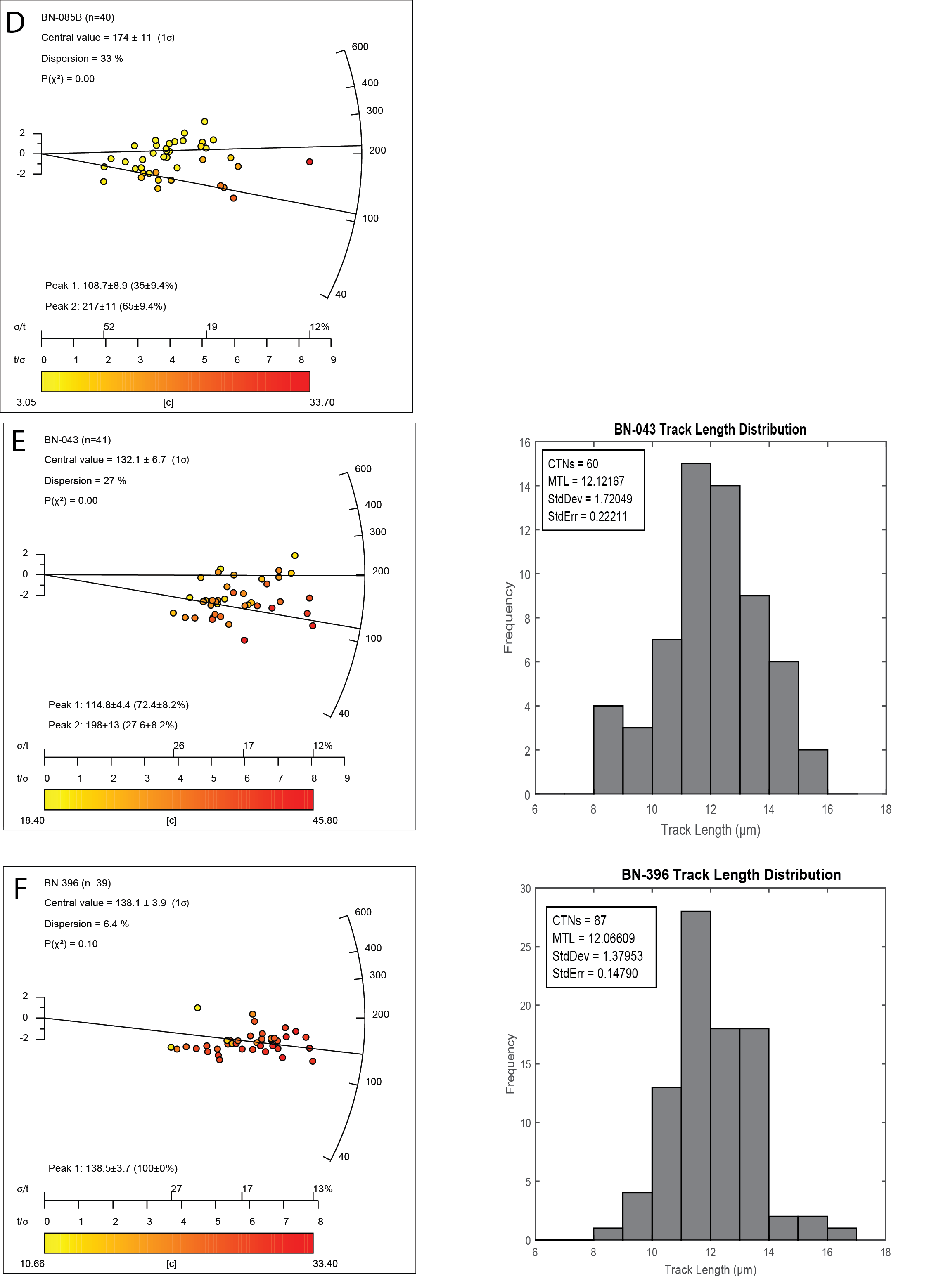


Supplementary Fig S3
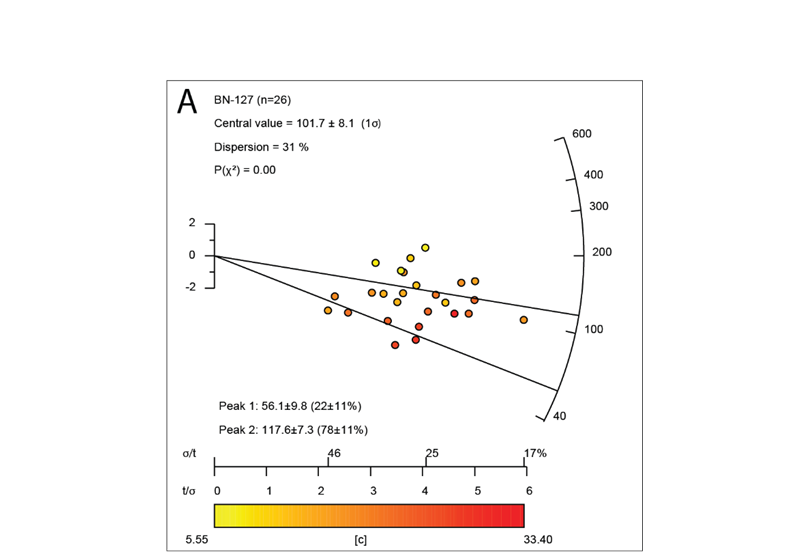


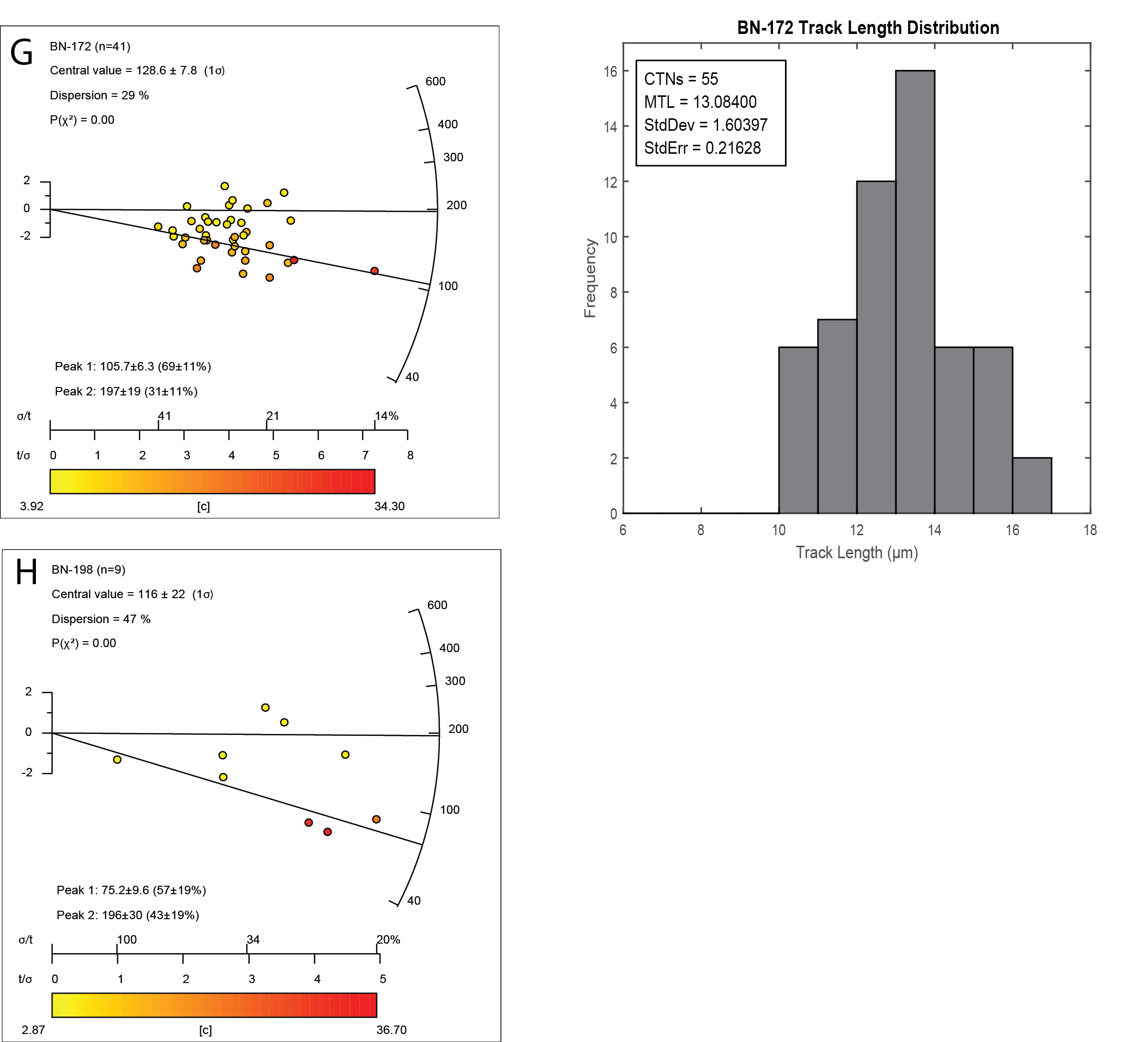

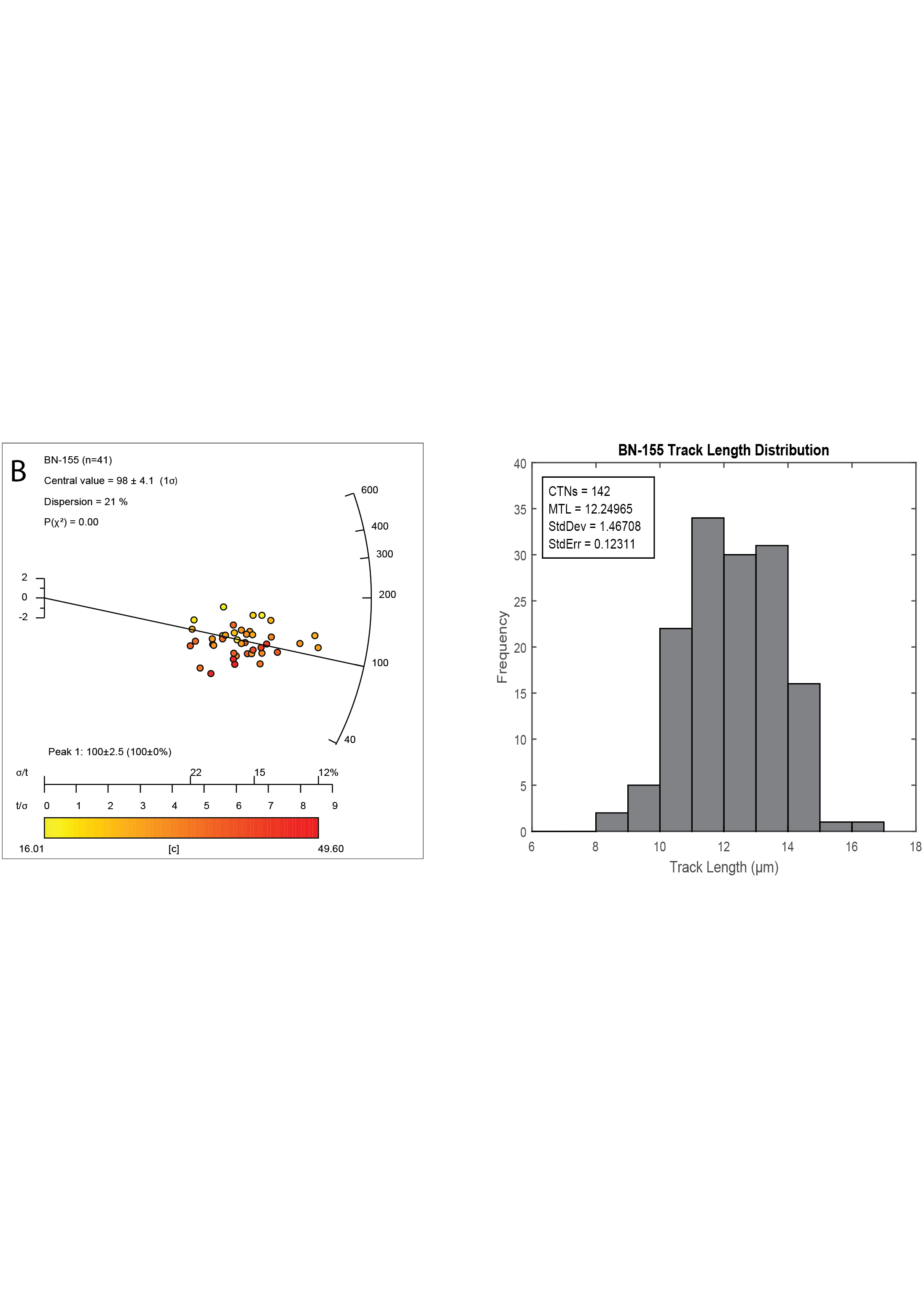

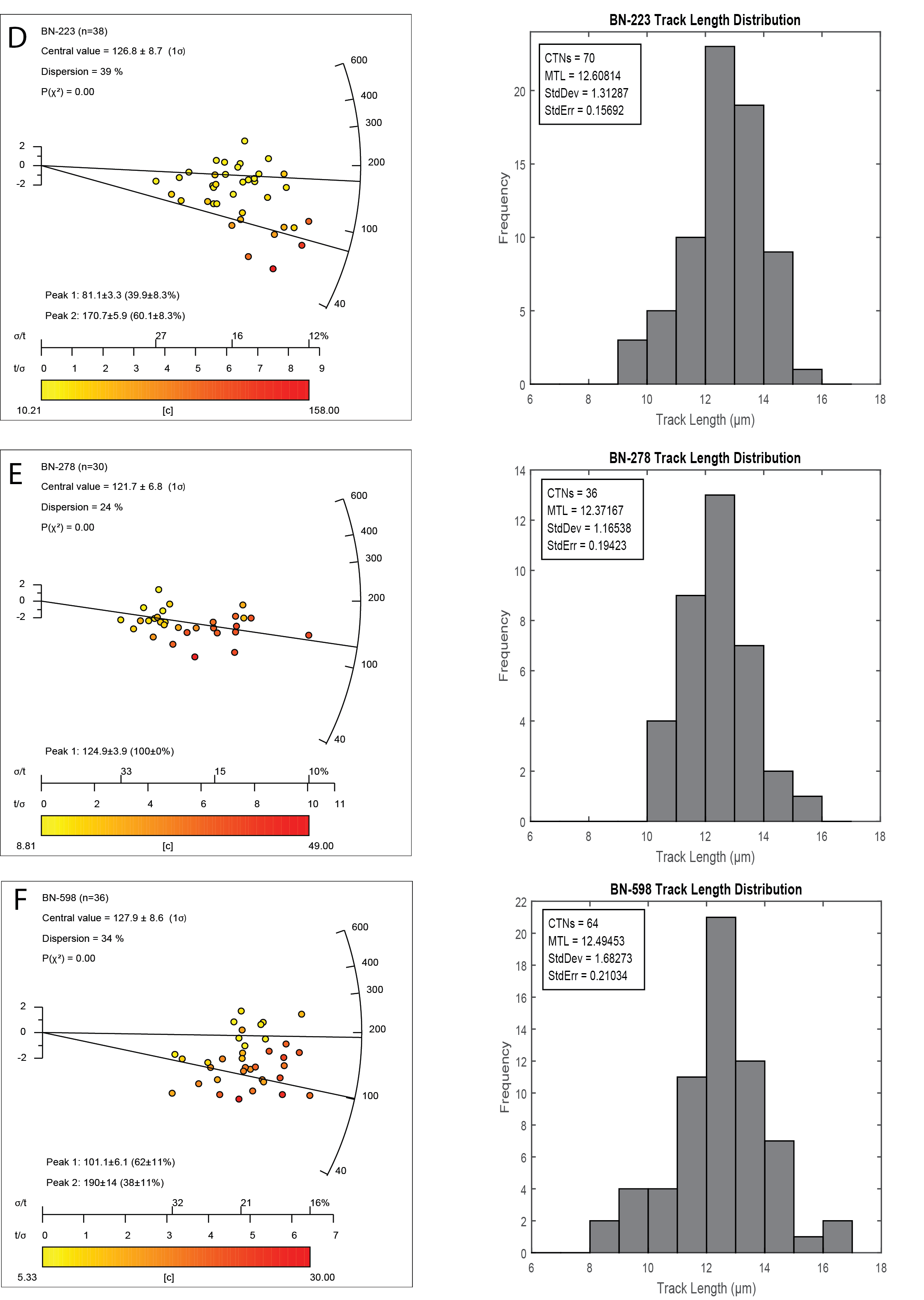


Supplementary Fig S4


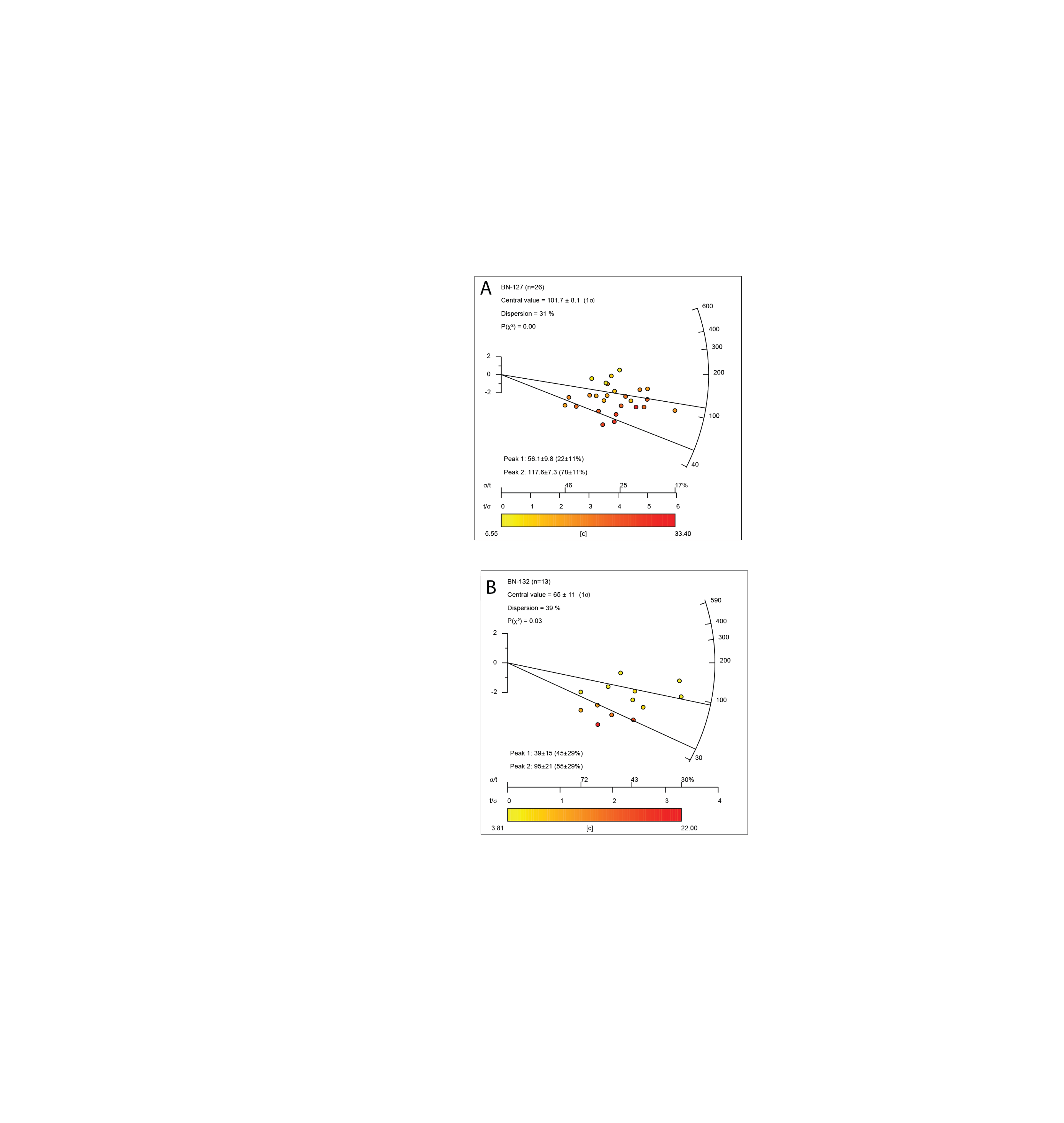


Supplementary Fig S5

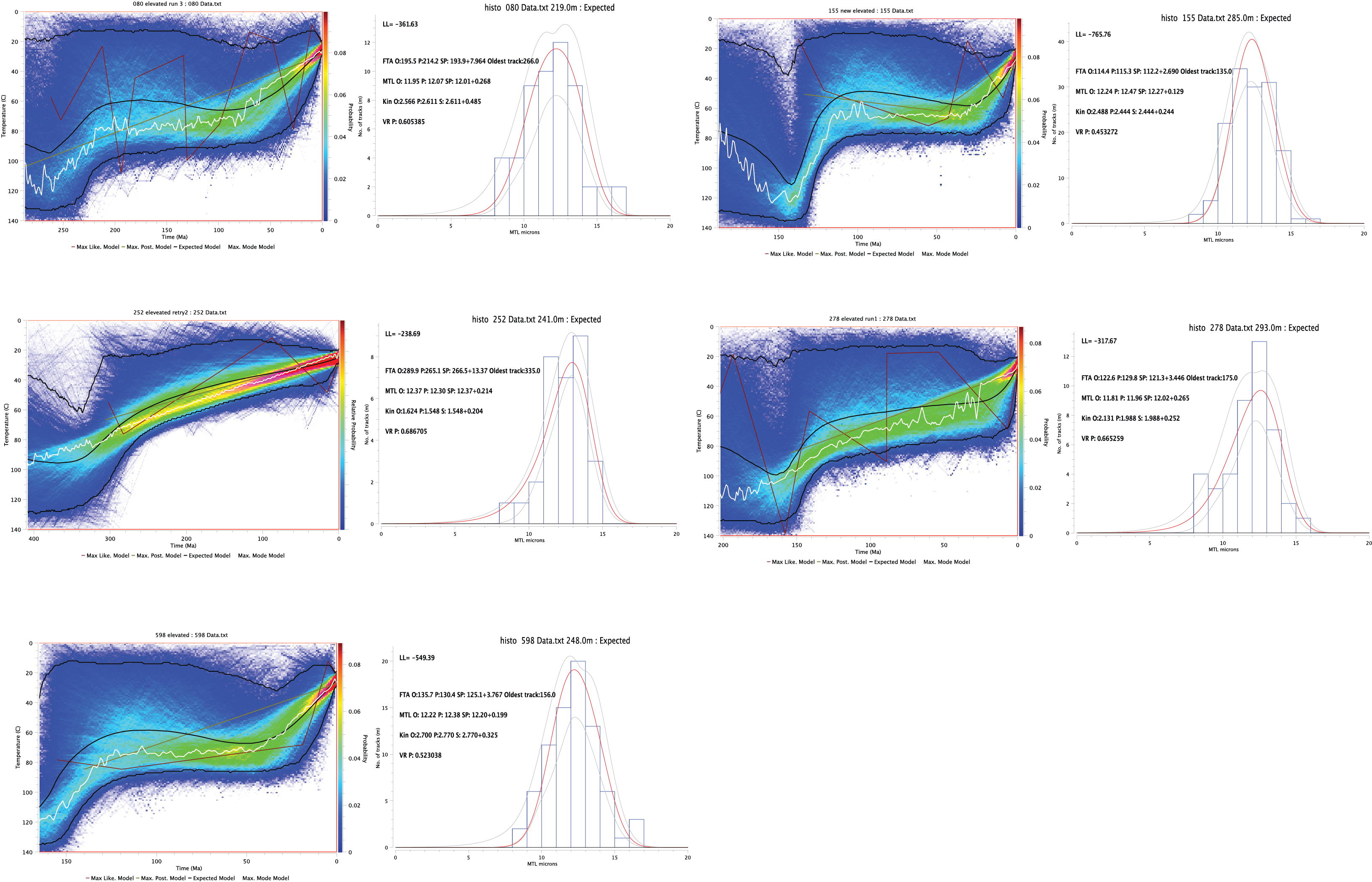


Supplementary Fig S6

| 1. **Thermochronologic Data** | | |  |  |  |  |  | |
| --- | --- | --- | --- | --- | --- | --- | --- | --- |
| *Samples and Data used in simulations* | | |  |  |  |  |  | |
|  | AFT | Data Source | All data needed for modelling published? | | | | Obs VS Pred | |
| AFT Ages |  |  |  |  |  |  | (Ma) | |
| 80 |  | Supplementary File | Yes |  |  |  | O:195.5-P:214.2 | |
| 252 |  | Supplementary File | Yes |  |  |  | O:289.9-P:265.1 | |
| 446 |  | Supplementary File | Yes |  |  |  | O:231.4-P:247.6 | |
| 16 |  | Supplementary File | Yes |  |  |  | O:176.7-P170.7 | |
| 43 |  | Supplementary File | Yes | Individual grain AFT ages | | | O:133.8-P:153.3 | |
| 396 |  | Supplementary File | Yes | required for modelling | | | O:136.9-P:143.5 | |
| 172 |  | Supplementary File | Yes | are included in | |  | O:130.4-P:124.1 | |
| 223 |  | Supplementary File | Yes | Supplementary File | |  | O:132-P:113 | |
| 278 |  | Supplementary File | Yes |  |  |  | O:122.6-P:129.8 | |
| 598 |  | Supplementary File | Yes |  |  |  | O:135.7-P:130.4 | |
| 155 |  | Supplementary File | Yes |  |  |  | O:114.4-P:115.3 | |
| AFT Lengths |  |  |  |  |  |  | (Um) | |
| 80 |  | Supplementary File | Yes |  |  |  | O:11.95–P:12.07 | |
| 252 |  | Supplementary File | Yes | Mean Track Lengths & Histograms O:12.37-P:12.30 | | | | |
| 446 |  | Supplementary File | Yes | for all samples provided in | | | O:11.62-P:11.59 | |
| 16 |  | Supplementary File | Yes | Supplementary File | |  | O:12.35-P:12.51 | |
| 43 |  | Supplementary File | Yes |  |  |  | O:11.65-P:11.64 | |
| 396 |  | Supplementary File | Yes |  |  |  | O:12.06-P:12.19 | |
| 172 |  | Supplementary File | Yes |  |  |  | O:12.93-P:13.02 | |
| 223 |  | Supplementary File | Yes |  |  |  | O:12.09-P:12.29 | |
| 278 |  | Supplementary File | Yes |  |  |  | O:11.81-P:11.96 | |
| 598 |  | Supplementary File | Yes |  |  |  | O:12.22-P12.38 | |
| 155 |  | Supplementary File | Yes |  |  |  | O:12.24-P:12.47 | |
|  |  |  |  |  |  |  |  | |
|  | = Thermochronometer used for modelling | | |  |  |  |  | |
|  |  |  |  |  |  |  |  | |
| *Data treatment, uncertainties and other relative constraints* | | | |  |  |  |  | |
| *AFT Data* |  |  |  |  |  |  |  | |
| *Compositional Model* | |  | Dpar (microns) | | No |  |  | |
| *Initial track length* | |  | 16.3um |  |  |  |  | |
| *Track length reduction standard* | | | 0.893 |  |  |  |  | |
| *Etchant* |  |  | 5.0 Molar |  |  |  |  | |
|  |  |  |  |  |  |  |  | |
| **2. Additional Geolgical Information** | | |  |  |  |  |  | |
| *Assumptions* |  |  |  | *Explanation* | |  |  | |
| *Present Day Temperature set to 25°C* | | |  |  |  |  |  |  |
| *Modelled elevations set according to present day sample elevation* | | | | |  |  |  |  |
| **3. System and Model specific parameters** | | |  |  |  |  |  |  |
| *FT Annealing model* | |  | Ketcham et al 2007 | |  |  |  |  |
| *Modelling Code* | |  | QTQt 5.6.0 PC | |  |  |  |  |
| *Statistical fitting criteria* | |  | Default QTQt Values | |  |  |  |  |
| *MCMC Parameters* | |  | Burn- in 150,000 and Post Burn-in = 150,000 | | | | |  |

Supplementary Table 2.
